# PINCH1 Promotes Fibroblast Migration in Extracellular Matrices and Influences Their Mechanophenotype

**DOI:** 10.3389/fcell.2022.869563

**Published:** 2022-05-16

**Authors:** Claudia Tanja Mierke, Alexander Hayn, Tony Fischer

**Affiliations:** Biological Physics Division, Faculty of Physics and Earth Science, Peter Debye Institute of Soft Matter Physics, Leipzig University, Leipzig, Germany

**Keywords:** cell mechanics, invasion, integrins, ILK, extracellular matrix, IPP complex, stiffness, fibroblasts

## Abstract

Cell migration performs a critical function in numerous physiological processes, including tissue homeostasis or wound healing after tissue injury, as well as pathological processes that include malignant progression of cancer. The efficiency of cell migration and invasion appears to be based on the mechano-phenotype of the cytoskeleton. The properties of the cytoskeleton depend on internal cytoskeletal and external environmental factors. A reason for this are connections between the cell and its local matrix microenvironment, which are established by cell-matrix adhesion receptors. Upon activation, focal adhesion proteins such as PINCH1 are recruited to sites where focal adhesions form. PINCH1 specifically couples through interactions with ILK, which binds to cell matrix receptors and the actomyosin cytoskeleton. However, the role of PINCH1 in cell mechanics regulating cellular motility in 3D collagen matrices is still unclear. PINCH1 is thought to facilitate 3D motility by regulating cellular mechanical properties, such as stiffness. In this study, PINCH1 wild-type and knock-out cells were examined for their ability to migrate in dense extracellular 3D matrices. Indeed, PINCH1 wild-type cells migrated more numerously and deeper in 3D matrices, compared to knock-out cells. Moreover, cellular deformability was determined, e.g., elastic modulus (stiffness). PINCH1 knock-out cells are more deformable (compliable) than PINCH1 wild-type cells. Migration of both PINCH1^−/−^ cells and PINCH1^fl/fl^ cells was decreased by Latrunculin A inhibition of actin polymerization, suggesting that actin cytoskeletal differences are not responsible for the discrepancy in invasiveness of the two cell types. However, the mechanical phenotype of PINCH1^−/−^ cells may be reflected by Latrunculin A treatment of PINCH1^fl/fl^ cells, as they exhibit resembling deformability to untreated PINCH1^−/−^ cells. Moreover, an apparent mismatch exists between the elongation of the long axis and the contraction of the short axis between PINCH1^fl/fl^ cells and PINCH1^−/−^ cells following Latrunculin A treatment. There is evidence of this indicating a shift in the proxy values for Poisson’s ratio in PINCH1^−/−^ cells compared with PINCH1^fl/fl^ cells. This is probably attributable to modifications in cytoskeletal architecture. The non-muscle myosin II inhibitor Blebbistatin also reduced the cell invasiveness in 3D extracellular matrices but instead caused a stiffening of the cells. Finally, PINCH1 is apparently essential for providing cellular mechanical stiffness through the actin cytoskeleton, which regulates 3D motility.

## Introduction

Cell migration and invasion relies on the interplay of cell-matrix adhesion molecules such as integrins, which send signals from the extracellular environment to the internal intracellular milieu of cells via integrin-linked kinase-1 (ILK-1). The particularly interesting new cysteine histidine-rich-protein1 (PINCH1, synonymously referred to as Lims1) is a non-catalytic protein possessing five double-zinc-finger LIM domains that tether to ILK-1 and other proteins, such as Nck2 (Kovalevich et al., 2011). At sites where focal adhesions form, known as adhesomes or intracellular integrin adhesion complex (IAC) (Winograd-Katz et al., 2014), the adaptor proteins PINCH1 and Parvin can interact with ILK-1 to assemble the heterotrimeric complex, referred to as ILK/PINCH/parvin (IPP) complex (Tu et al., 1998; Tu et al., 1999; Li et al., 1999; Nikolopoulos and Turner, 2000; Tu et al., 2001; Yamaji et al., 2001; Braun et al., 2003; Chu et al., 2006). These adaptor proteins bind in a direct manner to diverse cytoplasmic proteins, among them Nck2 for PINCH1 and filamentous actin for Parvin (Tu et al., 1998, 2001). Specifically, the ankyrin repeat domain of the third IPP component ILK couples to PINCH1 and its kinase domain connects to Parvin. Hence, ILK appears to function as a critical regulatory protein for IPP assembly. Finally, IAC serves as a platform to attract multiple proteins for assembly of focal adhesions and establishment of a signal transduction route that subsequently couples focal adhesions and actin cytoskeleton of cells (Tu et al., 2001; Wu, 2004; Legate et al., 2006; Wickström et al., 2010; Qin and Wu, 2012).

PINCH comprises two constituents, PINCH1 and PINCH2, with each constituent comprising five LIM domains. Both PINCH1 and PINCH2 lack a catalytic domain. These properties make them perfect adaptation molecules that convey the assembly of multiprotein compounds. When PINCH1 is eliminated globally in mice, they are lethal (Liang et al., 2005), whereas PINCH2-KO mice exhibit no evident phenotypes (Stanchi et al., 2005; Chen et al., 2008). PINCH1 is capable of attaching to actin and actin-binding proteins including EPLIN (Epithelial Protein Lost In Neoplasm) with its LIM domain (Karaköse et al., 2015). It is important to note that PINCH2-ILK and PINCH1-ILK interferences are reciprocally exclusive. PINCH2 overexpression markedly hampered PINCH1-ILK interactome and decreased cell propagation and migration. These findings clearly outline a novel nuclear and focal adhesion protein that accompanies ILK and highlight an integral function of PINCH2 in regulating PINCH1-ILK interference, cell form alteration, and migration (Zhang et al., 2002a). However, enhanced expression of PINCH2, an ILK-binding protein that is structurally similar to PINCH1, impaired the down-regulation of ILK and α-Parvin that has been induced through the loss of PINCH1; however, it failed to restore the survival signal transduction or altered cell morphology (Fukuda et al., 2003).

Force dependent signal transduction via the RhoA/Rock pathway is encouraged by the IPP complex that elevates the activity of non-muscle myosin II (Schiller et al., 2013). Apart from the coupling of the intracellular IAC to the actin cytoskeleton and the extracellular matrix, PINCH1 within the IPP complex may contribute to the regulation of F-actin dynamics (Calderwood et al., 2000; Geiger et al., 2001; Geiger et al., 2009), filopodia formation and the interplay between actin and myosin filaments. Subsequently through this actin-myosin filament interplay cellular mechanical characteristics, such as cell stiffness and viscoelasticity, may be impacted. In reaction to tension, the IPP complex has been considered to exert a developmentally antecedent role or function: It strengthens the attachment between integrins and extracellular matrix (Vakaloglou et al., 2016). However, in IPP complex mutants, the connection between integrin and extracellular matrix ruptures due to elevated muscle contraction in muscle attachment sites (Vakaloglou et al., 2016). From a mechanistic point of view, the IPP complex is necessary to transduce force-evoked cues that slow down the integrin turnover across the plasma membrane, so that the immobile integrin moiety is sufficient to sustain stresses. Moreover, the IPP complex encourages the bundling of actin that may also be associated with the actin cortex including the actin nucleators Formin and Arp2/3 (Laplaud et al., 2020). Specifically, actin-related protein 2 (ARP2) and ARP3 are both members of a complex that initiates the assembly of new actin filaments. The Arp2/3 complex represents a conserved actin nucleator made up of two actin-related proteins (ARP2 and ARP3) and another five complex-specific protein subunits (ARPC1 to ARPC5) (Welch et al., 1997). This complex prefers to induce new filaments from the face of existing filaments, which results in a ramified actin meshwork. In particular, the function of ARP2/3 is linked to the generation of planar, actin-controlled protrusions referred to as the well-known lamellipodia. In addition, the Arp2/3 complex can be found in dynamic puncta within filopodia and lamellipodia of propagating cells (Johnston et al., 2008).

To extend our understanding of the adhesion components and the IPP complex and subsequently the IACs, we seek to figure out the function of PINCH1 in cell migration and invasion and its contribution of the cellular mechanophenotype. Our hypothesis is that PINCH1 contributes to the function of the IPP complex as on the one hand it couples the actin cytoskeleton to the extracellular matrix in IACs and on the other hand provides a tension-based strengthening of the force sensing-dependent cell surface receptors, such as α 5 β1 integrins (Schiller et al., 2013). Therefore, we hypothesize that PINCH1 carries out a similar prominent function as ILK-1 that has been demonstrated to elevate motility of cells in 3D environments and the mechanical properties of cells, such as their stiffness (or reverse their deformability) (Kunschmann et al., 2017). In agreement with this hypothesis, we have also recently shown that PINCH1 responds differently to external mechanical force application using a magnetic tweezer device through α 5 β1 integrin bound fibronectin coated magnetizable beads such as that knock-down of PINCH1 softens the cells (Aermes et al., 2020). To explore the function of PINCH1 more comprehensively, we selected cells in which PINCH1 is knocked out, referred to as PINCH1^−/−^ mouse embryonic fibroblasts (MEFs) and control cells that possess PINCH1, referred to as PINCH1^fl/fl^ MEFs as model systems for this study. Western blot analysis of these cell lines confirmed that the PINCH1 protein is absent in PINCH1^−/−^ MEFs, whereas the PINCH2 protein is absent in PINCH1^fl/fl^ and PINCH1^−/−^ MEFs (Stanchi et al., 2005). Hence, when employing these two MEF cell types, only the effect of PINCH1 can be explored on cellular motility and mechanics.

We showed that PINCH1^−/−^ and PINCH1 wild-type fibroblasts exhibited different invasiveness in 3D matrices. In this regard, PINCH1^−/−^ cells manifested lower invasiveness. Moreover, in PINCH1^−/−^ cells optical cell stretching revealed increased deformability compared to PINCH1^fl/fl^ cells, suggesting that PINCH1 supports migration and invasion in 3D environments possibly by providing enhanced stiffness. It is supported by the findings that stiffer fibroblasts migrated more efficiently in 3D matrix confinements (Kunschmann et al., 2019). Moreover, it underpins the universal hypothesis that in general stiffer cells are more invasive in 3D environments. Finally, we showed that blocking of actin polymerization by Latrunculin A decreased the invasiveness of both PINCH1^−/−^ cells and PINCH1^fl/fl^ cells, thus indicating that alteration of the actin cytoskeleton cannot account for the differences in invasiveness of the two cell types. In fact, Latrunculin A exhibits an additive effect on impairing invasion in PINCH1 knockout cells. This implies that PINCH1 may operate by a different mechanism, otherwise an additional decrease in invasion would not be apparent when PINCH1 knock-out cells are treated with Latrunculin A. However, the mechanical phenotype of PINCH1^−/−^ cells can be mirrored by treatment of PINCH1^fl/fl^ cells with Latrunculin A, as these cells exhibit similar deformability as untreated PINCH1^−/−^ cells. Conversely, the blockage of myosin II with Blebbistatin impaired cellular invasiveness, whereas the deformability of both cell types decreased. This work contributes, firstly, to the understanding of the close connection between IACs and the extracellular matrix environment in fibroblasts and secondly, to the identification of a specific involvement of PINCH1, similar to that of ILK-1, in the functional roles of the IPP complex. Consequently, PINCH1 is capable of regulating the mechanosensitivity and mechanical characteristics of cells.

## Results

### PINCH1 Knock-Out Decreases the Migration of Cells in 3D Extracellular Matrices

**FIGURE 1:**
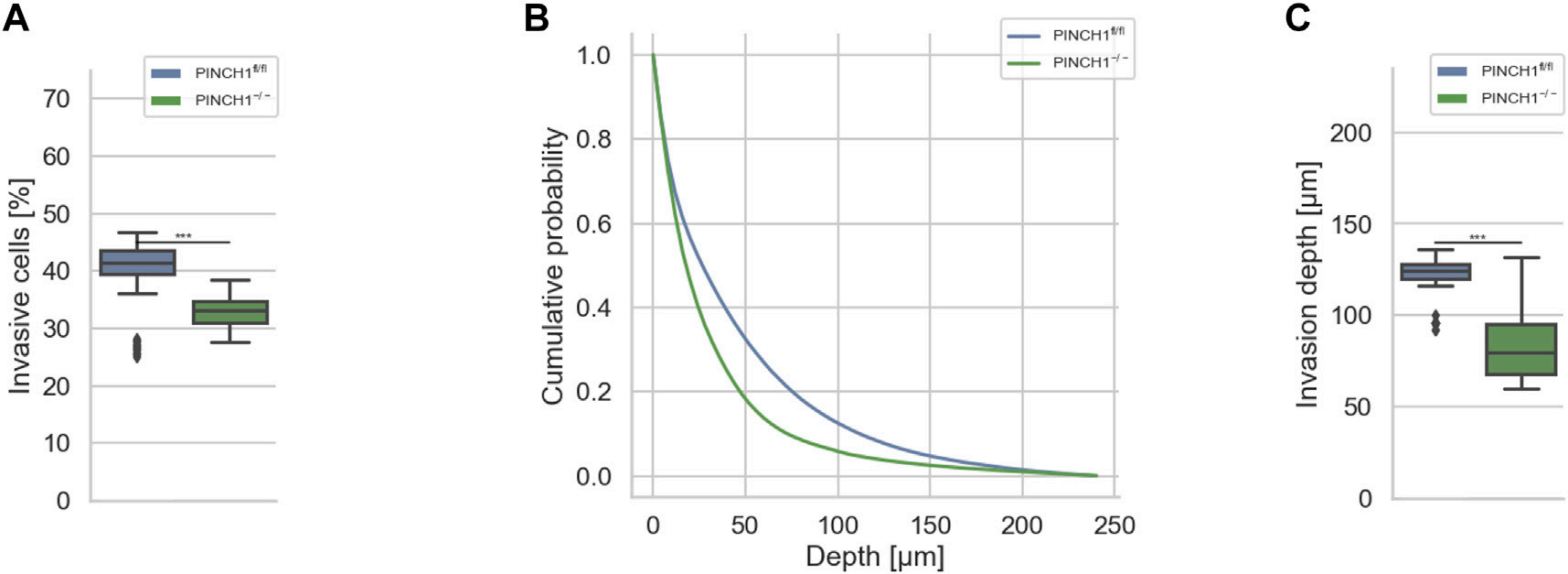
3D collagen invasion of PINCH1^−/−^ and PINCH1^fl/fl^ cells. **(A)** Average percentage of invasive migrating PINCH1^−/−^ cells (*n* = 813,241 total number of cells) is pronouncedly decreased compared to PINCH1^fl/fl^ cells (*n* = 1,336,561 total number of cells). **(B)** Invasion depth profiles of the invasive PINCH1^−/−^ cells (green) and invasive PINCH1^fl/fl^ cells (blue) that migrated in the 3D extracellular matrices after three days of culture at 37°C, 95% humidity and 5% CO_2_. **(C)** The average invasion depths of PINCH1^−/−^ cells (green column) is pronouncedly lower compared to PINCH1^fl/fl^ cells (blue column). **(A,C)** data are presented as box and whiskers plots (Q2 represent mean values). (***p < 0.001; Kruskal-Wallis test).

As PINCH1 forms a tricomplex with ILK and Parvin to couple the extracellular matrix scaffold through integrins, such as β 1 and β 3 (Wickström et al., 2010), to the actin cytoskeleton of cells, it seems likely that this coupling impacts cell migration and invasion in a 3D microenvironment system. In an experimental model, the extracellular matrix scaffold can simply be modeled through a commonly employed 3D collagen hydrogel. This hydrogel is composed of collagen I monomers from two different sources, namely fetal bovine skin and rat tail collagen. Both collagens were mixed in a mass ratio of 1:2 (rat/bovine), as we have employed this collagen mixture previously to explore the effect of ILK on the migration and invasion of cells (Kunschmann et al., 2017). This distinct combination of collagens is chosen as it provides a rather homogenous network that is capable to mimic the *in vivo* scaffold of connective tissue (Hayn et al., 2020). To explore the effect of PINCH1 on fibroblast migration and invasion we determined the migratory ability of PINCH1 knockout (PINCH1^−/−^) and PINCH1 wild-type (PINCH1^fl/fl^) fibroblasts by placing each on dense 3D extracellular matrices (with a thickness of approximately 500 μm and a collagen concentration of 3.0 g/l) and allowing them to invade (Figure 1). After three days, we analyzed the percentage of cells that were capable to invade (Figure 1A), measured their invasion profiles (Figure 1B) and assessed their invasion depths (Figure 1C). In fact, the invasiveness was pronouncedly lower in PINCH1^−/−^ compared to PINCH1^fl/fl^ cells. Specifically, the invasion profile of PINCH1^−/−^ cells (n = 813241 total number of cells, *n* = 4–5 repeats) was pronouncedly lower compared to PINCH1^fl/fl^ cells (*n* = 1,336,561 total number of cells) (Figure 1B). Consequently, the findings indicate that PINCH1 contributes to the invasive phenotype of cells.

### PINCH1 Knock-Out Reduces Cellular Stiffness and Modulates Relaxation Behavior of Strained Cells

**FIGURE 2:**
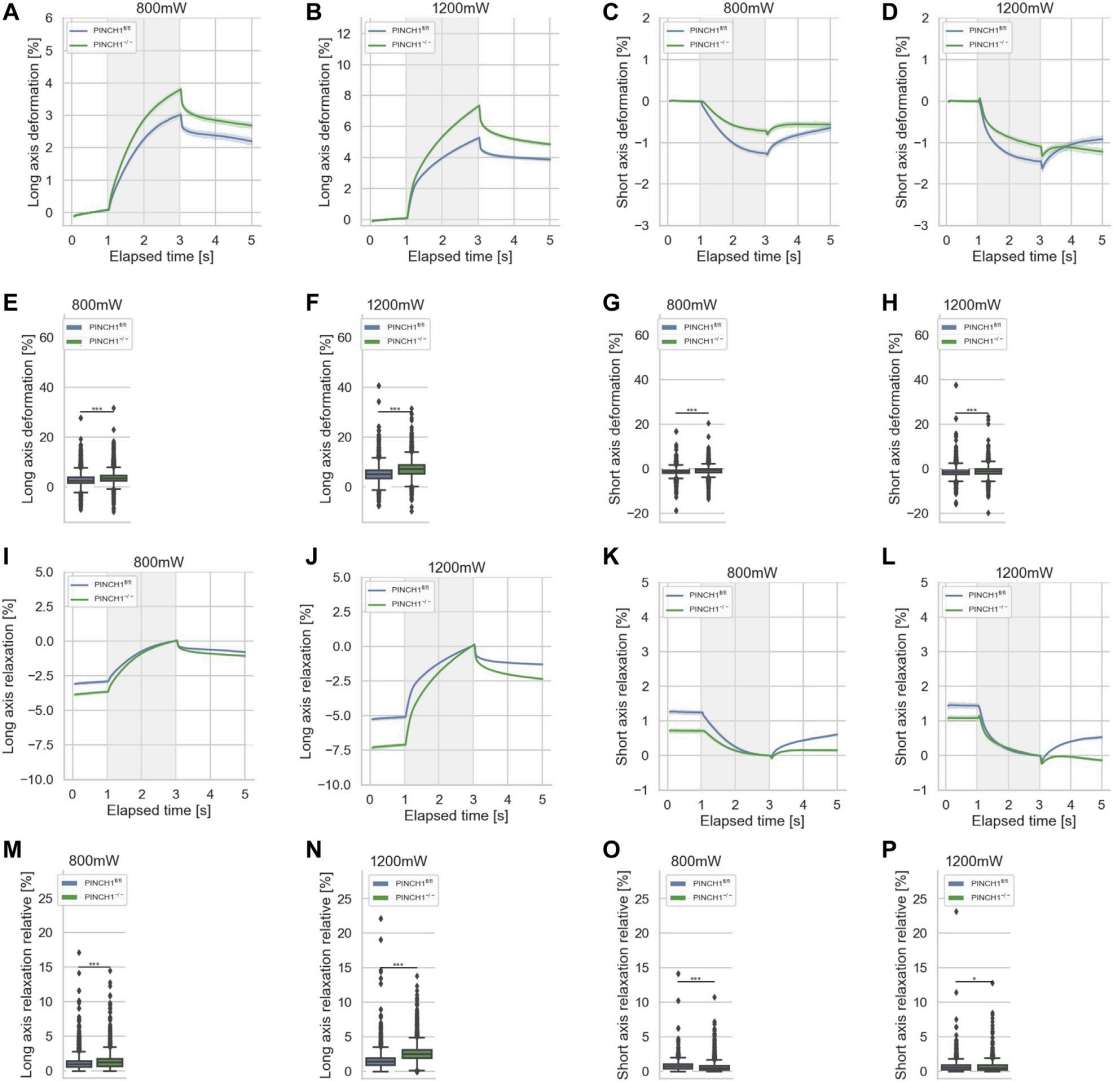
Cellular deformability of PINCH1^−/−^ (green) and PINCH1^fl/fl^ cells (blue) was measured using an optical cell stretcher. During the first second each cell is trapped, then stretched for 2 s with a middle laser power of 800 mW or a high laser power of 1,200 mW. The deformability curves of PINCH1^−/−^ cells and PINCH1^fl/fl^ cells at their long axis, which is parallel to the laser beams **(A,B)**, and short axis, which is perpendicular to the laser beams **(C,D)**. **(E,F)** Maximal deformability (at time point of 3 s) of PINCH1^−/−^ cells and PINCH1^fl/fl^ cells at laser powers of 800 mW (median values, *n* = 16,907 cells for PINCH1^−/−^ cells and *n* = 16,817 cells for PINCH1^fl/fl^ cells) to 1,200 mW (median values, *n* = 16,878 cells for PINCH1^−/−^ cells and *n* = 16,615 cells for PINCH1^fl/fl^ cells) at their long axis. **(G,H)** Maximal deformability of PINCH1^−/−^ and PINCH1^fl/fl^ cells at laser powers of 800–1,200 mW at their short axis. After the stretching process, the viscoelastic relaxation is observed for 2 s. Relaxation behavior curves of the long axis for PINCH1^−/−^ cells and PINCH1^fl/fl^ cells at laser powers of 800 mW **(I)** and 1,200 mW **(J)**. **(K,L)** Short axis relaxation curves of PINCH1^−/−^ cells and PINCH1^fl/fl^ cells at the two laser powers. **(M,N)** Maximal relaxation (at time point 3 s, where the deformation is maximal) of PINCH1^−/−^ cells and PINCH1^fl/fl^ cells at the two laser powers at their long axis. **(O,P)** Maximal relaxation of PINCH1^−/−^ and PINCH1^fl/fl^ cells at the two laser powers at their short axis. **(A–D, I–L)** data are presented as median values with a 95.0% confidence interval **(E–H, M–P)** data are presented as box and whiskers plots (Q2 represent median values). **(A–P)** The experiments have been conducted as 4 to 6 independent repetitions. (***p < 0.001, *p < 0.05; Kruskal-Wallis test).

It is known that mechanical characteristics of cells determine their migratory capacity, such as the percentage of invasive cells and their invasion depths (Gjorevski et al., 2015; Mierke et al., 2017; Fischer et al., 2020). Therefore, in order to assess the mechanical properties of cells, we analyzed the deformability (inverse stiffness) of singular PINCH1^−/−^ cells and compared them with the deformability of singular PINCH1^fl/fl^ cells employing an optical cell stretcher device. In general, changes in the deformation of the longitudinal axis are referred to as deformability of cells. This deformation is caused by forces acting on the surface of the individual cell by two opposing laser beams (Mierke, 2019). The deformation curves of the long axis of the two cell types showed that PINCH1^−/−^ cells were significantly softer (less stiff) compared to PINCH1^fl/fl^ cells for low and high laser powers applied for cell probing (Figures 2A,B; Supplementary Figure S1). Apart from the deformation of the long axis of the cells, we also assessed changes of the short axis, which is orthogonal to the laser beam axes (Figures 2C,D). It can be seen that PINCH1^−/−^cells were less deformed than PINCH1^fl/fl^ cells in terms of short axis deformation for both laser powers (Figures 2C,D). The maximal deformations at the 3 s timepoint are provided for the long and short axes at the two laser powers (Figures 2E–H). The deformation behavior of the long and short axes was more severe at the higher laser power (1,200 mW) compared to the lower laser power (800 mW), which was observed for both cell types.

The relaxation is plotted relative to the maximal deformation at the end of the stretch phase. After the removal of the stretching force by turning down the laser power to the trapping power of 100 mW, cells relaxed for 2 s and their elongation at the long axis was reduced (Figures 2I,J). In addition, the compressive strain of the short axis was reduced at both low and high laser powers, as relaxation is observed at both. (Figures 2K,L). Relative cell relaxations are provided at the 5 s time point for both the long axis (Figures 2M,N) and the short axis (Figures 2O,P). Consequently, these findings elucidate that PINCH1 knock-out reduces the stiffness of fibroblasts indicating a function of PINCH1 in the mechanophenotype of cells.

### PINCH1 Knock-Out Exhibits Impaired Invasiveness After Latrunculin A Treatment and Altered Cell Shape Before/After Latrunculin A Treatment

**FIGURE 3:**
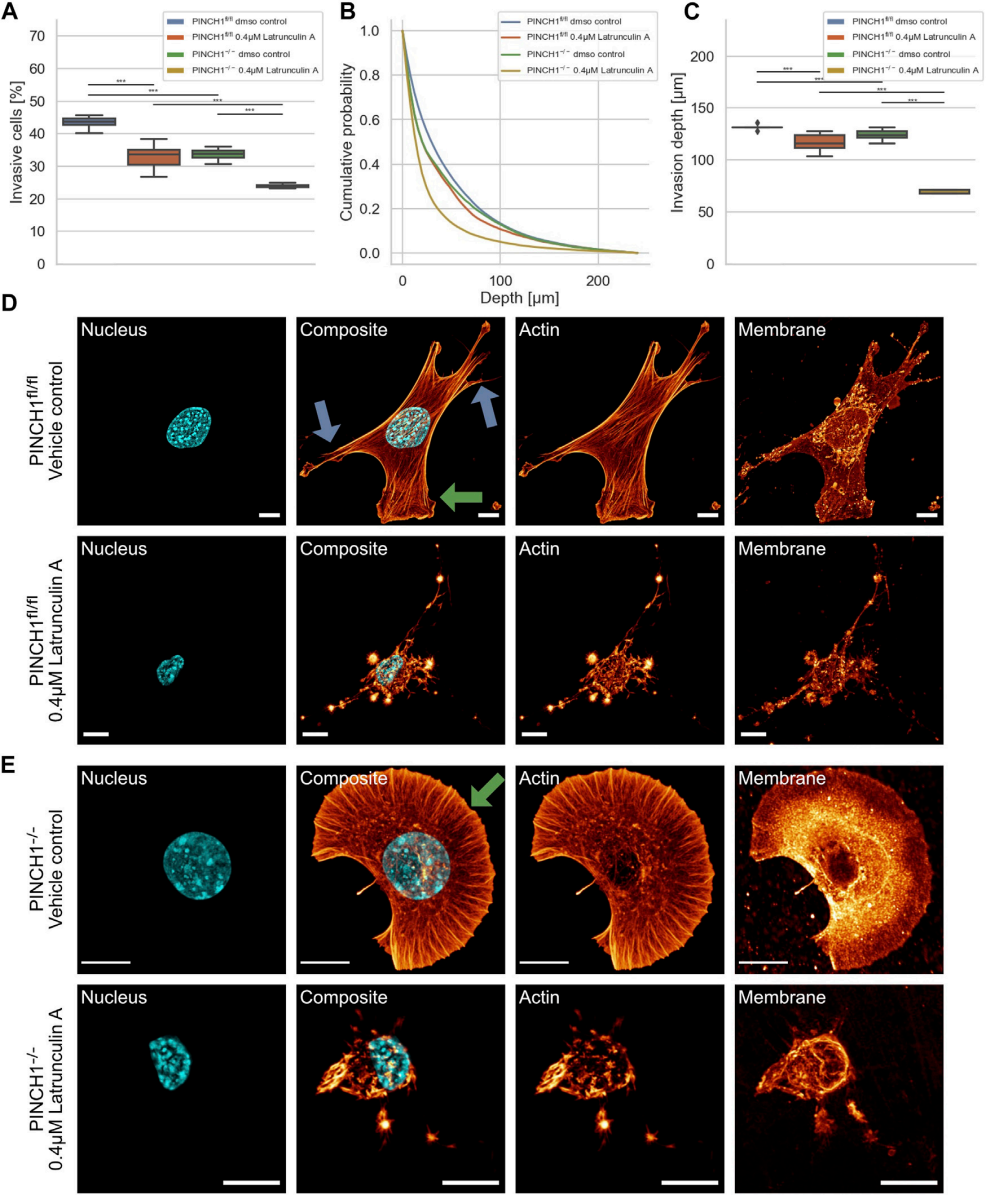
3D collagen invasion of PINCH1^−/−^ and PINCH1^fl/fl^ cells in the presence or absence of Latrunculin A treatment. **(A)** Average percentage of invasive migrating PINCH1^−/−^ cells (yellow, *n* = 982,978 cells with Latrunculin A; green, *n* = 312,813 buffer-treated control cells) is pronouncedly decreased compared to PINCH1^fl/fl^ cells (red, *n* = 307,743 cells with Latrunculin A; blue, *n* = 1,135,294 buffer-treated control cells) after Latrunculin A. The Latrunculin A-treatment caused a reduction in the percentage of invasive cells compared to their buffer-treated controls. **(B)** Invasion depth profiles of the invasive PINCH1^−/−^ cells (green, *n* = 105,835 cells in the absence, yellow, *n* = 236,225 in presence of Latrunculin A and invasive PINCH1^fl/fl^ cells (blue, *n* = 495,742 cells in the absence, red, n = 101,896 in presence of Latrunculin A that migrated in the 3D extracellular matrices after three days of culture at 37°C, 95% humidity and 5% CO_2_ in the presence and absence of Latrunculin A. **(C)** The invasion depths of Latrunculin A-treated PINCH1^−/−^ cells is most pronouncedly impaired compared to Latrunculin A-treated PINCH1^fl/fl^ cells. Moreover, the average invasion depths of PINCH1^−/−^ cells (green column) is pronouncedly lower compared to PINCH1^fl/fl^ cells (blue column) either in the presence or absence of Latrunculin A. **(A,C)** data are presented as box and whiskers plots (Q2 represent mean values). **(D,E)** Confocal laser scanning images are presented as maximal projections. Inhibition of actin polymerization by Latrunculin A alters the morphology and the actin cytoskeleton of PINCH1^fl/fl^ cells **(D)** and PINCH1^−/−^ cells **(E)**. Nuclear shape, composite of nuclear shape, actin cytoskeleton and membrane shape of a representative PINCH1^fl/fl^ cell **(D)** or PINCH1^−/-^ cell **(E)** in absence (upper row) and presence of Latrunculin A (lower row) is provided. **(D,E)** Blue arrows indicate lamellipodia, green arrows indicate filopodia. All experiments are conducted in quadruple or quintuple. All scale bars are 10 µm. (***p < 0.001; Kruskal-Wallis test).

To explore whether the effect of PINCH1 on fibroblast migration and invasion involves the actin cytoskeleton, we determined the migratory ability of PINCH1^−/−^ cells and PINCH1^fl/fl^ cells by placing each of the two cell types on dense 3D extracellular matrices in the presence or absence of 0.4 µM Latrunculin A (Figures 3A–C). As expected, the invasiveness of the two cell types was decreased in terms of percentage of invasive cells and invasion depths of cells (Figures 3A–C). The invasion depth was significantly reduced in PINCH1^−/−^ cells after inhibition of actin polymerization by Latrunculin A (Figure 3C).

There is ample evidence and agreement that cells utilize their actin cytoskeleton to promote and elevate their movement in 3D environments. For a correlation of decreased invasiveness and altered cellular mechanical properties under inhibition of actin polymerization with the cell shape, we investigated the effect of Latrunculin A on the morphology and actin cytoskeleton of PINCH1^fl/fl^ and PINCH1^−/−^ cells. Therefore, we cultured both cell types on planar substrates coated with 10 μg/ml laminin and treated either with DMSO or 0.4 μM Latrunculin A for 2 h. After fixing the cells, they were stained with Alexa Fluor 546 phalloidin, Hoechst 33342 and DiD. Confocal laser scanning microscopy was employed to reveal the actin cytoskeleton and morphology of the cells by recording z-stacks with a z-distance between neighboring images of approximately 130–200 nm. We observed that Latrunculin A impaired protrusion formation, such as lamellipodia and filopodia in PINCH1^−/−^ cells to an extent similar to PINCH1^fl/fl^ cells in all fields of view (Figures 3D,E, see arrows). Moreover, PINCH1^−/−^ cells (Figure 3E) and PINCH1^fl/fl^ cells (Figure 3D) displayed a less branched actin network or no obviously detectable actin fibers. These findings indicate that the actin cytoskeletal filament network seems to be critical for providing cellular invasion in 3D matrix confinements. Based on the altered cell morphology and actin cytoskeleton after Latrunculin A treatment of the two cell types it seems to be reasonable that their mechanical characteristics were changed.

### PINCH1 Knock-Out Treatment With Latrunculin A Increases Cellular Deformability

**FIGURE 4:**
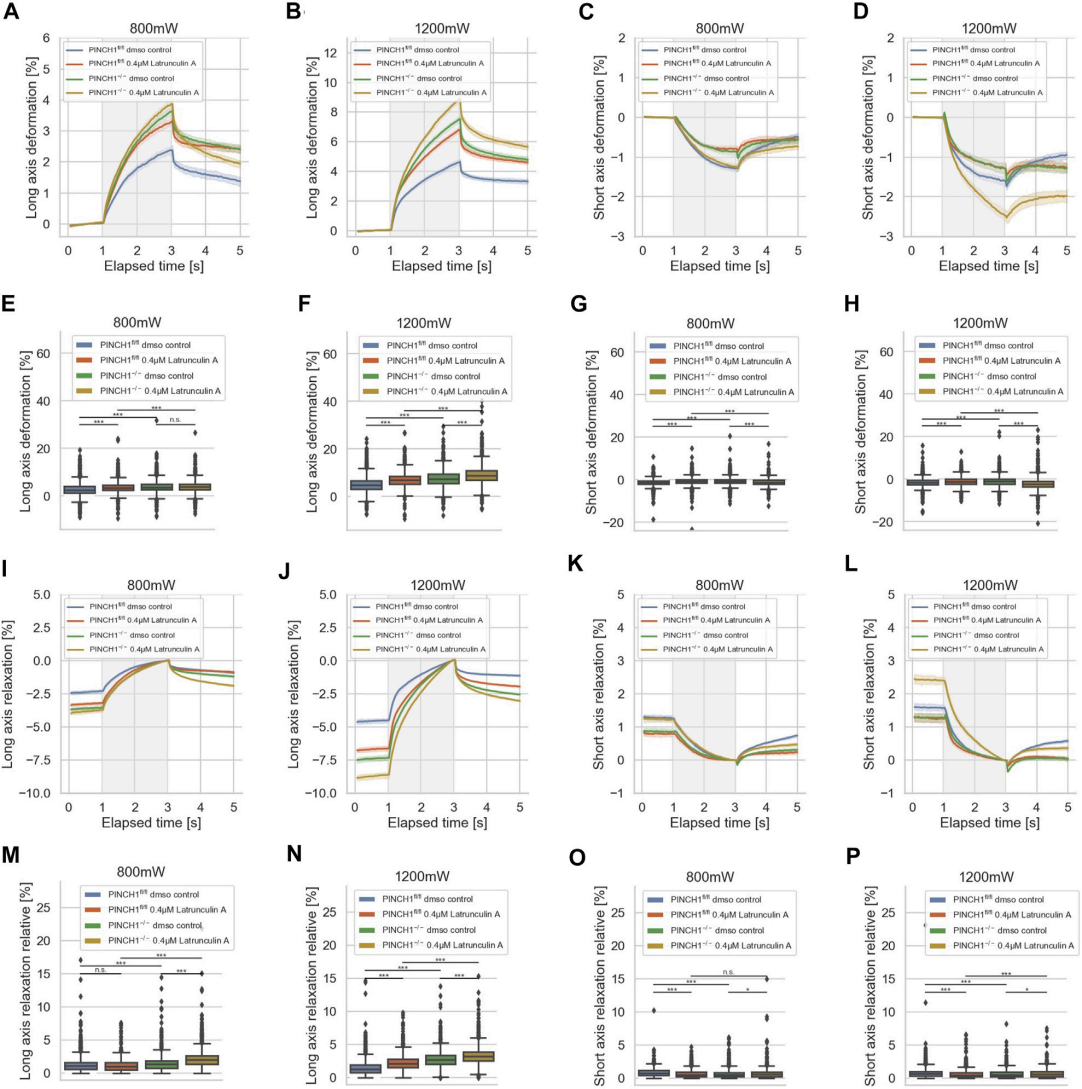
Effect of Latrunculin A on cellular deformability. Cellular deformability of PINCH1^−/−^ cells and PINCH1^fl/fl^ cells were measured at 800 mW laser power in the presence (median values, *n* = 1,683 cells for PINCH1^−/−^ cells (yellow) and *n* = 1,630 cells for PINCH1^fl/fl^ cells (red), respectively) or absence of Latrunculin A (median values, *n* = 1,596 cells for PINCH1^−/−^ cells (green) and *n* = 1,641 cells for PINCH1^fl/fl^ cells (blue) and at 1,200 mW in the presence of Latrunculin A (median values, *n* = 1,778 cells for PINCH1^−/−^ cells (yellow) and *n* = 1,697 cells for PINCH1^fl/fl^ cells (red)) or in the absence of Latrunculin A (median values, *n* = 1,583 cells for PINCH1^−/−^ cells (green) and *n* = 1,687 cells for PINCH1^fl/fl^ cells (blue) using an optical cell stretcher. The deformability curves of PINCH1^−/−^ cells and PINCH1^fl/fl^ cells at their long axis **(A,B)**, and short axis **(C,D)** with/without Latrunculin A treatment. **(E–H)** Maximal deformability of PINCH1^−/−^ cells and PINCH1^fl/fl^ cells at laser powers of 800–1,200 mW at their long **(E,F)** and short axis **(G,H)** in the presence or absence of Latrunculin A treatment. After the stretching process, the viscoelastic relaxation is observed for 2 s **(I–P)**. Relaxation behavior curves of the long axis for PINCH1^−/−^ cells and PINCH1^fl/fl^ cells at laser powers of 800 mW **(I)** and 1,200 mW **(J)** with/without Latrunculin A treatment. **(K,L)** Short axis relaxation curves of PINCH1^−/−^ cells and PINCH1^fl/fl^ cells at the two laser powers with/without Latrunculin A treatment. **(M,N)** Maximal relaxation (at time point 3 s, where the deformation is maximal) of PINCH1^−/−^ cells and PINCH1^fl/fl^ cells at the two laser powers at their long axis with/without Latrunculin A treatment. **(O,P)** Maximal relaxation of PINCH1^−/−^ and PINCH1^fl/fl^ cells at the two laser powers at their short axis with/without Latrunculin A treatment. **(A–D, I–L)** Data are presented as median values with a 95.0% confidence interval, **(E–H, M–P)** data are presented as box and whiskers plots (Q2 represent median values). **(A–P)** The experiments have been conducted as 4 to 6 independent repetitions. (***p < 0.001, *p < 0.05, n.s. = not significant; Kruskal-Wallis test).

Since the invasiveness of cells, such as fibroblasts, may be due to the polymerization of actin, it can be assumed that the mechanical properties of the cells are also altered, e.g., that the cells become softer after treatment with Latrunculin A. Moreover, the expression of PINCH1 is known to be associated with the actin polymerization, such as the formation of actin reticular networks through the Arp2/3 complex. Specifically, we aimed to determine whether there were differences in the reaction to Latrunculin A of PINCH1^−/−^ and PINCH1^fl/fl^ cells that could be attributed to the presence of PINCH1. To reveal whether the migratory differences between the two cell types rely on the actin cytoskeleton, we treated the two cell types with Latrunculin A and determined their deformability using the optical cell stretcher (Figure 4). After treatment of PINCH1 knockout cells with Latrunculin A, the deformation of the cells was enhanced by elongation along their longitudinal axis, which was more pronounced at 1,200 mW than at 800 mW. (Figures 4A,B). Moreover, there is a discrepancy between long axis elongation and short axis contraction between PINCH1^fl/fl^ cells and PINCH1^−/−^ cells (Figures 4A–D). This indicates a change in the proxy for the Poisson’s ratio in the PINCH1^−/−^ cells compared to the PINCH1^fl/fl^ cells (Supplementary Figure S2). This is likely due to alterations in cytoskeletal architecture as seen in 2D on laminin, such as the different protrusions (see arrows). Therefore, we considered graphing the relative long versus short axis deformation as a surrogate for Poisson’s ratio. In fact, PINCH1 knock-out cells show a smaller proxy for the Poisson’s ratio compared to PINCH1^fl/fl^ cells, and the addition of Latrunculin A reduced the proxy for the Poisson’s ratio of WT cells to approximately that of null cells (Supplementary Figure S2). In addition, we found that Latrunculin A treatment resulted to a similar change in the relative long axis and short axis deformation in PINCH1^fl/fl^ cells. The contraction of the short axis of PINCH1^−/−^ cells after Latrunculin A treatment was enhanced at a laser power of 800 mW and even stronger at a laser power of 1,200 mW (Figures 4C,D). This behavior is extremely evident in the maximum deformability of the cells for their long axis (Figures 4E,F) and their short axis (Figures 4G,H). These findings indicate that the Latrunculin A-treated PINCH1^fl/fl^ cells resemble the deformability properties of untreated PINCH1^−/−^ cells at both laser powers for the long and short axes of the cells (Figures 4E–H).

After returning the laser power to 100 mW (trapping power), a relaxation behavior of the two cell types and all conditions were observed for both cell axes, indicating that the deformability is at least partly reversable (Figures 4I–P). Maximal relaxation values (Figures 4M–P) point in the same direction as the entire relaxation curves (Figures 4I–L).

### PINCH1 Knock-Out Exhibits Impaired Invasiveness and Altered Cell Shape After Blebbistatin Treatment

**FIGURE 5:**
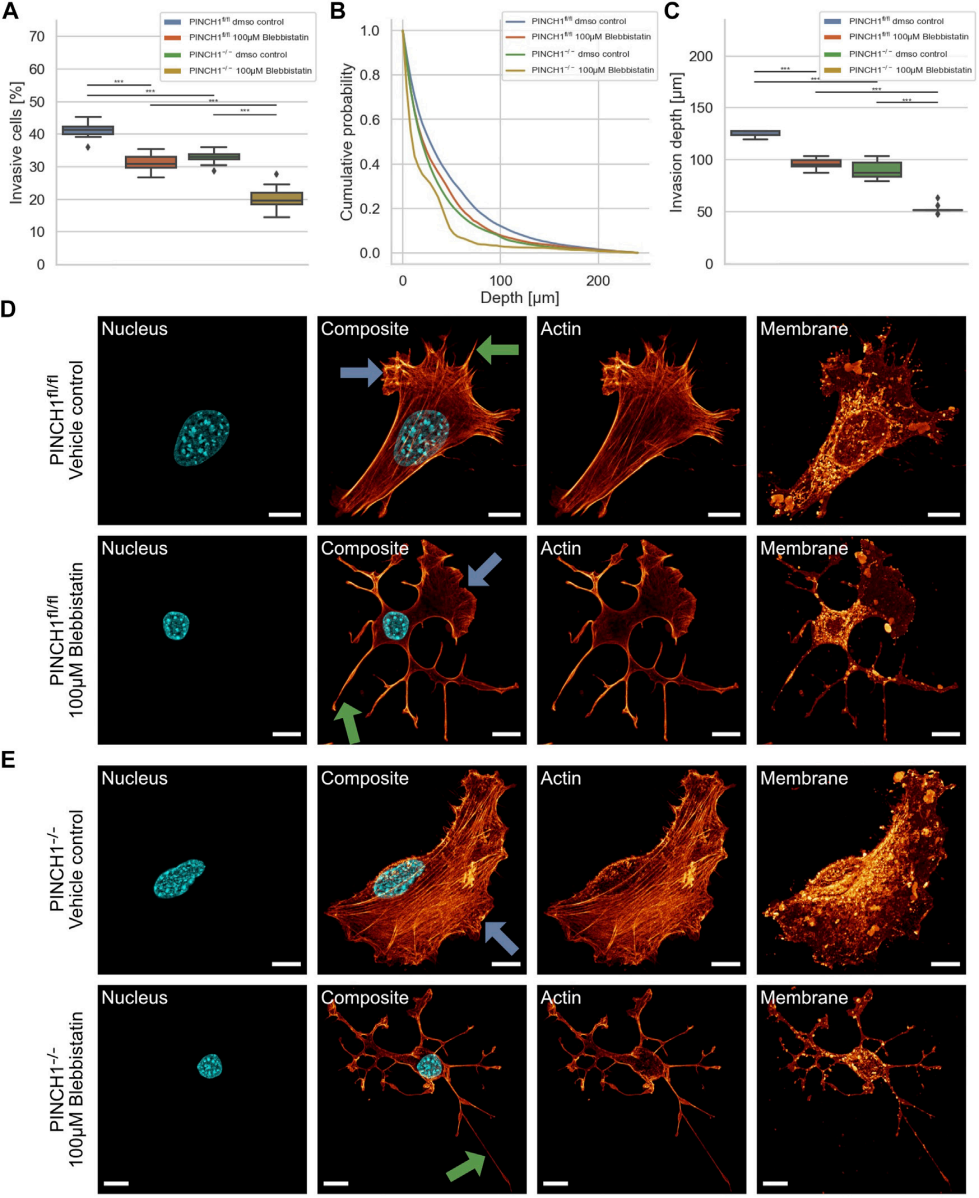
3D collagen invasion of PINCH1^−/−^ and PINCH1^fl/fl^ cells in the presence or absence of Blebbistatin treatment. **(A)** Average percentage of invasive migrating PINCH1^−/−^ cells (yellow, *n* = 52,398 cells with Blebbistatin; green, *n* = 1,136,648 buffer-treated control cells) is pronouncedly decreased compared to PINCH1^fl/fl^ cells (red, *n* = 200,927 cells with Blebbistatin; green, *n* = 591,980 buffer-treated control cells) after Blebbistatin treatment. The Blebbistatin-treatment caused a reduction in the percentage of invasive cells compared to their buffer-treated controls. **(B)** Invasion depth profiles of the invasive PINCH1^−/−^ cells (green, *n* = 374,151 cells in the absence, *n* = 10,723 cells in the presence of Blebbistatin) and invasive PINCH1^fl/fl^ cells (blue, *n* = 243,534 cells in the absence, *n* = 62,641 cells in the presence of Blebbistatin) that migrated in the 3D extracellular matrices after three days of culture at 37°C, 95% humidity and 5% CO_2_ in the presence and absence of Blebbistatin. **(C)** The invasion depth of Blebbistatin-treated PINCH1^−/−^ cells is most pronouncedly impaired compared to blebbistatin-PINCH1^fl/fl^ cells. Moreover, the average invasion depth of PINCH1^−/−^ cells (green column) is pronouncedly lower compared to PINCH1^fl/fl^ cells (blue column) either in the presence or absence of Blebbistatin. **(A,C)** data are presented as box and whiskers plots (Q2 represent mean values). **(D,E)** Confocal laser scanning images are presented as maximal projections. Inhibition of actin polymerization by Blebbistatin alters the morphology and the actin cytoskeleton of PINCH1^fl/fl^ cells **(D)** and PINCH1^−/−^ cells **(E)** using a confocal laser scanning microscopy. Cells were cultured on planar substrates coated with 10 μg/ml laminin and treated for 2 h with Blebbistatin (100 μM). After fixation, the cells were stained with Alexa Fluor 546 Phallodin, Hoechst and DiD. Nuclear shape, composite of nuclear shape and action cytoskeleton, actin cytoskeleton and membrane shape of a representative PINCH1^fl/fl^ cell **(D)** or PINCH1^−/−^ cell **(E)** in absence (upper row) and presence of Blebbistatin (lower row) is provided. **(D,E)** Blue arrows indicate lamellipodia, green arrows indicate filopodia. All experiments are conducted in quadruple or quintuple. All scale bars are 10 μm. (***p < 0.001; Kruskal-Wallis test).

Cells can utilize contractile forces based on the interplay between actin and myosin II filaments to promote and elevate their movement in 3D environments. To explore whether the effect of PINCH1 on fibroblast migration and invasion involves myosin II filaments, we determined the migratory ability of PINCH1^−/−^ cells and PINCH1^fl/fl^ cells by placing each of them on dense 3D extracellular matrices in the presence or absence of 100 μM Blebbistatin (Figures 5A–C). As anticipated, the invasiveness of the two cell types was decreased in terms of percentage of invasive cells and invasion depth of cells (Figures 5A–C). Specifically, the invasion depths were pronouncedly impaired in PINCH1^−/−^ cells after inhibition of myosin IIA by Blebbistatin (Figure 5C).

For a correlation of decreased invasiveness and altered cellular deformation under inhibition of myosin II with the cell shape, we investigated the effect of Blebbistatin on the morphology and actin cytoskeleton of PINCH1^fl/fl^ and PINCH1^−/−^ cells. Therefore, the two cell types were treated with Blebbistatin and stained as described in Figure 3. We observed that Blebbistatin impaired protrusion formation, such as lamellipodia and filopodia in PINCH1^−/−^ cells to an extent similar to PINCH1^fl/fl^ cells in all fields of view (Figures 5D,E). Moreover, PINCH1^−/−^ cells (Figure 5E) and PINCH1^fl/fl^ cells (Figure 5D) displayed a weaker actin scaffold or no obviously detectable actin fibers. These findings indicate that the myosin filament network seems to be critical for providing cellular invasion and shape in 3D matrix confinements.

### PINCH1^fl/fl^ Cell Treatment With Blebbistatin Decreases Cellular Deformability

**FIGURE 6:**
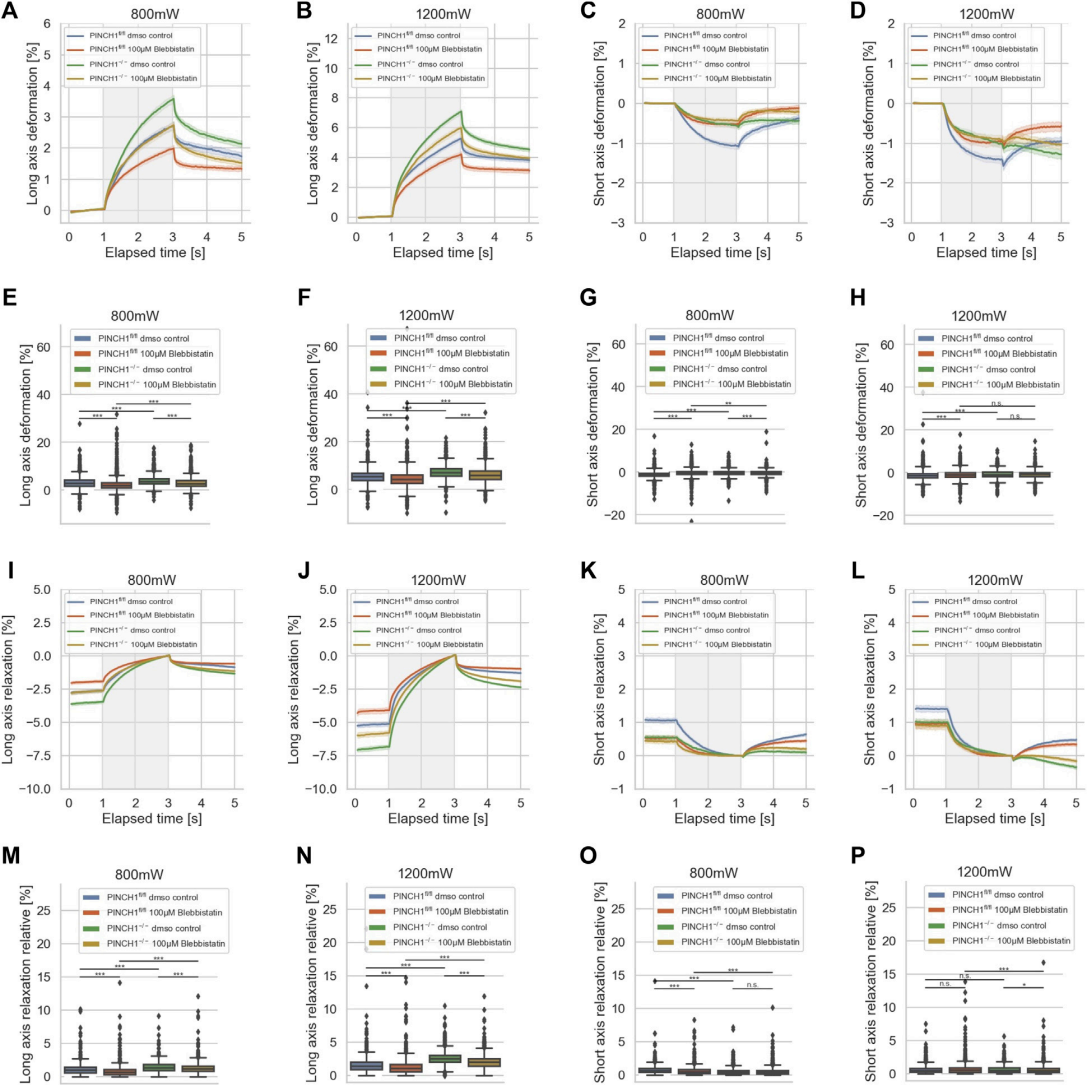
Impact of Blebbistatin on the deformability of cells. Cellular deformability of PINCH1^−/−^ cells and PINCH1^fl/fl^ cells were measured in the presence of Blebbistatin at 800 mW (median values, *n* = 1,657 cells for PINCH1^−/−^ cells (yellow) and *n* = 1,568 cells for PINCH1^fl/fl^ cells (red)) and at 1,200 mW (median values, *n* = 1,633 cells for PINCH1^−/−^ cells (green) and *n* = 1,498 cells for PINCH1^fl/fl^ cells (blue)) and in the absence of Blebbistatin at 800 mW (median values, *n* = 1,708 cells for PINCH1^−/−^ cells (green) and *n* = 1,706 cells for PINCH1^fl/fl^ cells (blue), respectively) and at 1,200 mW (median values, *n* = 1,744 cells for PINCH1^−/−^ cells (green) and *n* = 1,656 cells for PINCH1^fl/fl^ cells (blue)) using an optical cell stretcher. The deformability curves of PINCH1^−/−^ cells and PINCH1^fl/fl^ cells at their long axis **(A,B)**, and short axis **(C,D)** with/without Blebbistatin treatment. Maximal deformability of PINCH1^−/−^ cells and PINCH1^fl/fl^ cells at laser powers of 800–1,200 mW at their long **(E,F)** and short axis **(G,H)** in the presence or absence of Blebbistatin treatment. After the stretching process, the viscoelastic relaxation is observed for 2 s **(I–P)**. Relaxation behavior curves of the long axis for PINCH1^−/−^ cells and PINCH1^fl/fl^ cells at laser powers of 800 mW **(I)** and 1,200 mW **(J)** with/without Blebbistatin treatment. **(K,L)** Short axis relaxation curves of PINCH1^−/−^ cells and PINCH1^fl/fl^ cells at the two laser powers with/without Blebbistatin treatment. **(M,N)** Maximal relaxation (at time point 3 s, where the deformation is maximal) of PINCH1^−/−^ cells and PINCH1^fl/fl^ cells at the two laser powers at their long axis with/without Blebbistatin treatment. **(O,P)** Maximal relaxation of PINCH1^−/−^ and PINCH1^fl/fl^ cells at the two laser powers at their short axis with/without Blebbistatin treatment. **(A–D, I–L)** Data are presented as median values with a 95.0% confidence interval **(E–H, M–P)** data are presented as box and whiskers plots (Q2 represent median values). **(A–P)** The experiments have been conducted as 4 to 6 independent repetitions. (***p < 0.001, ** *p* < 0.01, * *p* < 0.05, n.s. = not significant; Kruskal-Wallis test).

As the invasiveness relies on the interplay between actin filaments and myosin II filaments, it can be assumed that additionally the mechanical characteristics of the cells were altered, such as that the cells get stiffer after treatment with Blebbistatin when the viscous sliding of myosin and actin filaments is impaired. Moreover, the expression of PINCH1 is known to be associated with the generation of forces via signaling from the IPP complex underneath integrins through RhoA/Rock that elevates the myosin II activity. Specifically, we sought to reveal whether there are differences in the reaction toward Blebbistatin that is attributable to the presence of PINCH1, hence we analyzed PINCH1^−/−^ and PINCH1^fl/fl^ cells. To figure out whether the migratory differences between the two cell types rely on the myosin-based forces, we treated the two cell types with Blebbistatin and determined their deformability using the optical cell stretcher (Figure 6). After treatment of PINCH1 knock-down cells with Blebbistatin, the deformation of the cells along their longitudinal axis was decreased, which was more pronounced after increasing the laser power from 800 to 1,200 mW for the stretching step (Figures 6A,B). The short axis of PINCH1^−/−^ cells after Blebbistatin treatment was impaired for the 800 mW laser power and less decreased for the 1,200 mW laser power (Figures 6C,D). The PINCH1^fl/fl^ cells displayed impaired deformability of their long axis at both laser powers (Figures 6A,B). Their short axis exhibited a decreased length after Blebbistatin treatment (Figures 6C,D). These behaviors were also seen at the maximal deformability of the PINCH1^−/−^ cells and PINCH1^fl/fl^ cells for their long axes (Figures 6E,F) and their short axes (Figures 6G,H). These findings indicate that PINCH1 knock-down renders the cells slightly less attainable to myosin inhibition. Moreover, the Blebbistatin-treated PINCH1^fl/fl^ cells and PINCH1^−/−^ cells resemble a less deformable mechanophenotype at both laser powers for the long and short axes of the cells (Figures 6E–H), indicating that the elastic modulus is increased (Supplementary Figure S1A). However, the differences in the deformability of the two cell types still remain.

After returning the laser power to that of the trapping step of 100 mW, a relaxation behavior of the two cell types and all conditions were observed for both cell axes, indicating that the deformability was at least partly reversable (Figures 6I–P). Maximal relaxation values (Figure 6M–P) point in the same direction as the entire relaxation curves (Figure 6I–L). Finally, it can be hypothesized that myosin motor-based contractility reduces the elastic component in the mechanophenotype of cells, whereas the impairment of myosin motors leads to elevated elasticity.

### Impairment of the Arp2/3 Complex Alters Invasion

**FIGURE 7:**
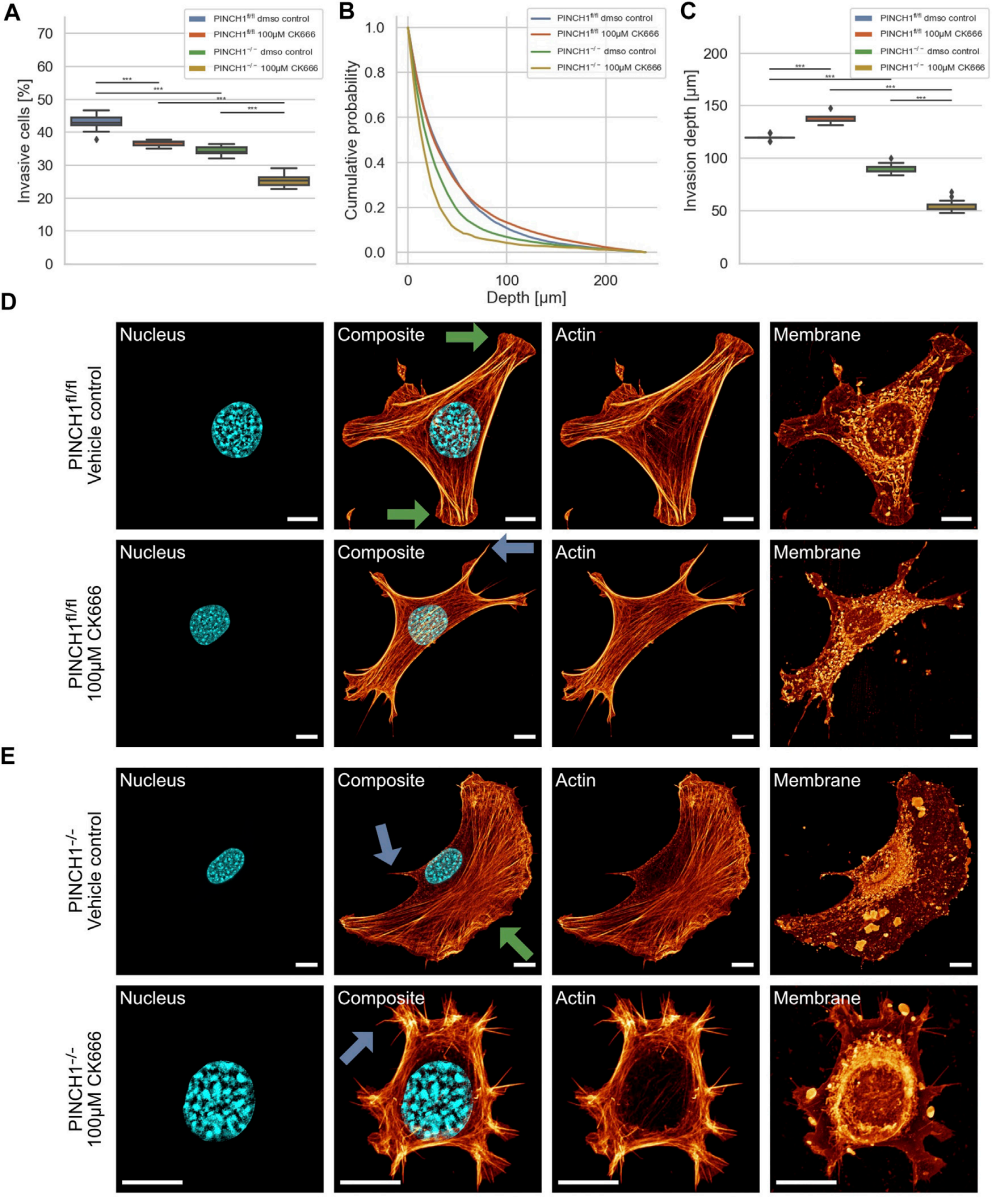
3D collagen invasion of PINCH1^−/−^ and PINCH1^fl/fl^ cells in the presence of absence of CK666 treatment. **(A)** Average percentage of invasive migrating PINCH1^−/−^cells (yellow, *n* = 46,199 cells with CK666; green, *n* = 435,330 buffer-treated control cells) is pronouncedly decreased compared to PINCH1^fl/fl^ cells (red, *n* = 538,616 cells with CK666; green, n = 322,738 buffer-treated control cells) after CK666. The CK666-treatment caused a reduction in the percentage of invasive cells compared to their buffer-treated controls. **(B)** Invasion depth profiles of the invasive PINCH1^−/−^ cells (green, n = 341,487 cells in the absence, yellow, *n* = 52,325 cells in the presence of CK666) and invasive PINCH1^fl/fl^ cells (blue, *n* = 338,771 cells in the absence, red, n = 141,729 cells in the presence of CK666) that were migrated in the 3D extracellular matrices after three days of culture at 37°C, 95% humidity and 5% CO_2_ in the presence and absence of CK666. **(C)** The invasion depths of CK666-treated PINCH1^−/−^ cells is most pronouncedly impaired compared to CK666-PINCH1^fl/fl^ cells. Moreover, the average invasion depths of PINCH1^−/−^ cells (green column) is pronouncedly lower compared to PINCH1^fl/fl^ cells (blue column) either in the presence or absence of CK666. **(A,C)** data are presented as box and whiskers plots (Q2 represent mean values). **(D,E)** Confocal laser scanning images are presented as maximal projections. Inhibition of the Arp2/3 complex by CK666 alters the morphology and the actin cytoskeleton of PINCH1^fl/fl^ cells **(D)** and PINCH1^−/−^ cells **(E)** using a confocal laser scanning microscopy. Cells were cultured on planar substrates coated with 10 μg/ml laminin and treated for 2 hours with CK666. After fixation, the cells were stained with Alexa Fluor 546 phallodin, Hoechst and DiD. Nuclear shape, composite of nuclear shape and action cytoskeleton, actin cytoskeleton and membrane shape of a representative PINCH1^fl/fl^ cell **(D)** or PINCH1^−/−^ cell **(E)** in absence (upper row) and presence of CK666 (lower row) is provided. **(D,E)** Blue arrows indicate lamellipodia, green arrows indicate filopodia. All experiments are conducted in quadruple or quintuple. All scale bars are 10 μm. (***p < 0.001; Kruskal-Wallis test).

To explore whether the effect of PINCH1 on fibroblast migration and invasion involves the branching of the actin cytoskeleton, we determined the migratory ability of PINCH1^−/−^ cells and PINCH1^fl/fl^ cells by placing each of the two cell types on dense 3D extracellular matrices in the presence or absence of 100 μM of the Arp2/3 inhibitor CK666 (Figures 7A–C). As hypothesized, the invasiveness of the two cell types was decreased in terms of percentage of invasive cells (Figures 7A,B). However, the invasion depth was severely reduced in PINCH1^−/−^ cells after inhibition of the Arp2/3 complex by CK666, whereas it was increased in CK666-treated PINCH1^fl/fl^ cells, indicating an interaction between PINCH1 and Arp2/3 function (Figure 7C).

There is ample evidence that cells utilize their branched actin network to promote and elevate their movement in 3D environments. For a correlation of decreased invasiveness and altered cellular deformation under inhibition of the Arp2/3 complex with the cell shape, we investigated the effect of CK666 on the morphology and actin cytoskeleton of PINCH1^fl/fl^ and PINCH1^−/−^ cells. Therefore, we cultured both cell types on planar substrates coated with 10 μg/ml laminin and treated either with 100 μM CK666 for 2 h. After fixing the cells, they were stained with Alexa Fluor 546 phalloidin, Hoechst 33342 and DiD. We observed that CK666 impaired protrusion formation, such as lamellipodia and filopodia in PINCH1^−/−^ cells to an extent similar to PINCH1^fl/fl^ cells in all fields of view (see arrows in Figures 7D,E). Moreover, PINCH1^−/−^ cells (Figure 7E) and PINCH1^fl/fl^ cells (Figure 7D) displayed a less branched actin network. These findings indicate that the actin cytoskeletal filament network seems to be critical for providing cellular invasion in 3D matrix confinements. Finally, the Arp2/3 complex appears to play an important role in determining the depth of invasion of PINCH1^fl/fl^ cells, as the depth of invasion is even greater when the Arp2/3 complex is impaired.

### Effect of the Arp2/3 Complex on Cell Mechanics

**FIGURE 8:**
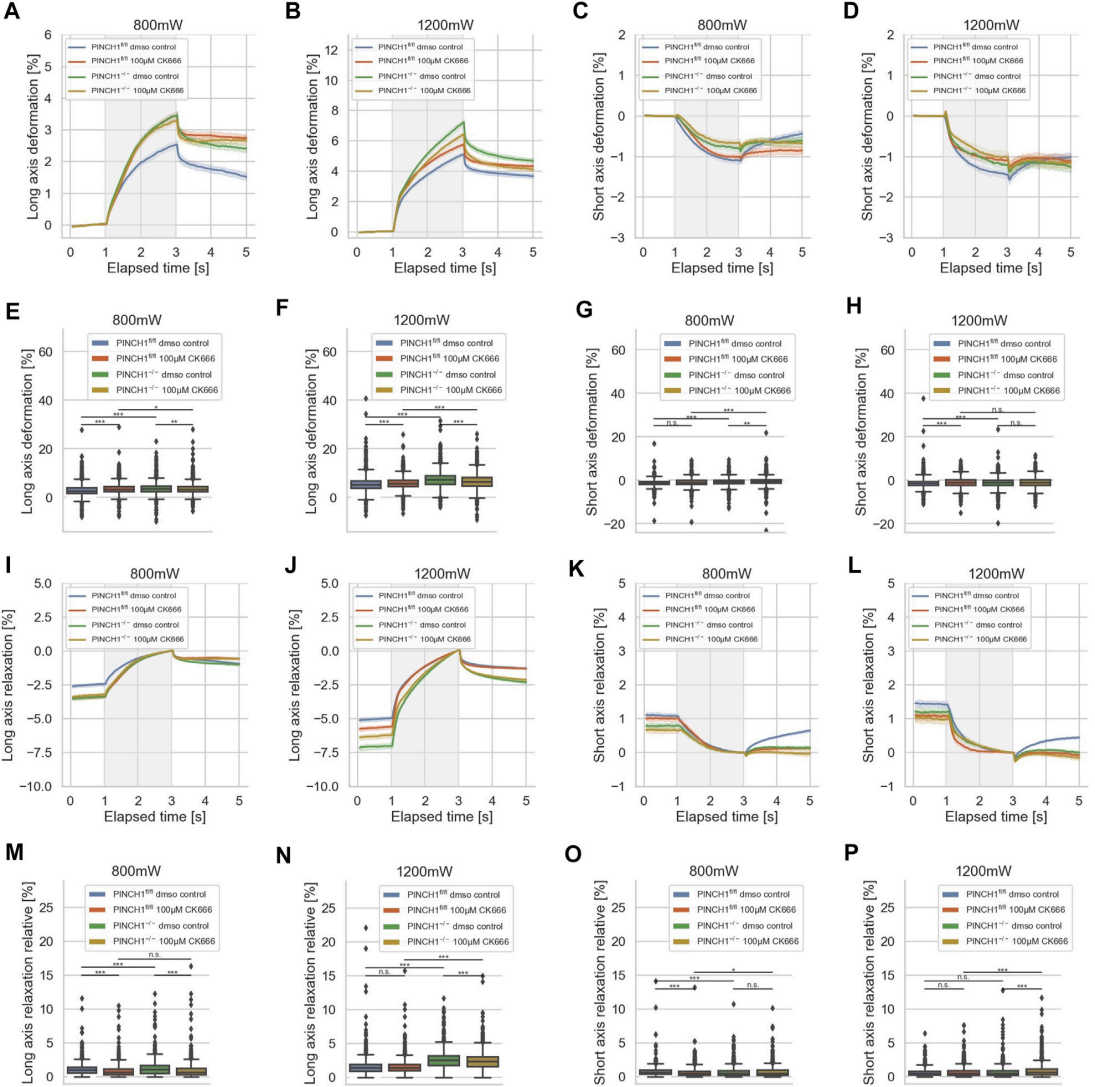
Effect of CK666 on cellular deformability. Cellular deformability of PINCH1^−/−^ cells and PINCH1^fl/fl^ cells were measured at 800 mW laser power in the presence (median values, *n* = 1,683 cells for PINCH1^−/−^ cells (yellow) and *n* = 1,630 cells for PINCH1^fl/fl^ cells (red)) of absence of CK666 (median values, *n* = 1,596 cells for PINCH1^−/−^ cells (green) and *n* = 1,641 cells for PINCH1^fl/fl^ cells (blue), respectively and 1,200 mW (median values, *n* = 1,778 cells for PINCH1^−/−^ cells (yellow) and *n* = 1,697 cells for PINCH1^fl/fl^ cells (red)) of absence of CK666 (median values, *n* = 1,583 cells for PINCH1^−/−^ cells (green) and *n* = 1,687 cells for PINCH1^fl/fl^ cells (blue) using an optical cell stretcher. The deformability curves of PINCH1^−/−^ cells and PINCH1^fl/fl^ cells at their long axis **(A,B)**, and short axis **(C,D)** with/without CK666 treatment. **(E,F)** Maximal deformability of PINCH1^−/−^ cells and PINCH1^fl/fl^ cells at laser powers of 800–1,200 mW at their long **(E,F)** and short **(G,H)** axis in the presence or absence of CK666 treatment. After the stretching process, the viscoelastic relaxation is observed for 2 s **(I–P)**. Relaxation behavior curves of the long axis for PINCH1^−/−^ cells and PINCH1^fl/fl^ cells at laser powers of 800 mW **(I)** and 1,200 mW **(J)** with/without CK666 treatment. **(K,L)** Short axis relaxation curves of PINCH1^−/−^ cells and PINCH1^fl/fl^ cells at the two laser powers with/without CK666 treatment. **(M,N)** Maximal relaxation (at time point 3 s, where the deformation is maximal) of PINCH1^−/−^ cells and PINCH1^fl/fl^ cells at the two laser powers at their long axis with/without CK666 treatment. **(O,P)** Maximal relaxation of PINCH1^−/−^ and PINCH1^fl/fl^ cells at the two laser powers at their short axis with/without CK666 treatment. **(A–D, I–L)** Data are presented as median values with a 95.0% confidence interval **(E–H, M–P)** data are presented as box and whiskers plots (Q2 represent median values). **(A–P)** The experiments have been conducted as 4 to 6 independent repetitions. (***p < 0.001, **p < 0.01, *p < 0.05, n.s. = not significant; Kruskal-Wallis test).

As the invasiveness relies on the branching of actin filaments, it can be assumed that additionally the mechanical characteristics of the cells were altered, such as that the cells get softer after treatment with CK666. Moreover, PINCH1 expression is known to be associated with the branching of the actin cytoskeletal network and its further polymerization. Specifically, we seek to reveal whether there are differences in the reaction toward the impairment of the Arp2/3 complex by CK666 of PINCH1^−/−^ and PINCH1^fl/fl^ cells that is attributable to the presence of PINCH1. To reveal whether the migratory differences between the two cell types rely on the branching of the actin cytoskeleton via Arp2/3, we treated the two cell types with CK666 and determined their deformability using the optical cell stretcher (Figure 8). After CK666 treatment of PINCH1 knock-out cells, the deformation of the cells along their longitudinal axis was reduced and their elastic modulus was increased (Supplementary Figure S1A), which was more pronounced using a higher laser power of 1,200 mW compared to the lower laser power of 800 mW for the stretching step, where the difference was rather small (Figures 8A,B). However, the CK666 treatment of PINCH1^fl/fl^ cells led to elevated deformability at both low and high laser powers (Figures 8A,B).

The decrease of the short axis deformation of PINCH1^−/−^ cells after CK666 treatment was enhanced for the 800 mW laser power and even more elevated for the 1,200 mW laser power (Figures 8C,D). This behavior is also seen at the maximal deformability of the PINCH1^−/−^ cells for their long axis (Figures 8E,F) and their short axis (Figures 8G,H). These findings indicate that PINCH1 knock-out renders the cells more attainable to actin branching impairment. Moreover, the CK666-treated PINCH1^fl/fl^ cells resemble the deformability properties of untreated PINCH1^−/−^ cells at both laser powers for the long and short axes of the cells (Figures 8E–H).

After returning the laser power to that of the trapping step of 100 mW, a relaxation behavior of the two cell types and all conditions were observed for both cell axes, indicating that the deformability was at least partly reversable (Figures 8I–P). Maximal relaxation values (Figures 8M–P) point in the same direction as the entire relaxation curves (Figures 8I–L). In line with the deformation behavior, the CK666-treated PINCH1^fl/fl^ cells resemble the relaxation properties of untreated PINCH1^−/−^ cells at both laser powers for the long and short axes of the cells (Figures 8I–P). These findings indicate that the crosslinked cytoskeleton, such as Arp2/3 branching of actin filaments, provides more actomyosin filament sliding and hence more friction that leads to an enhanced viscous part (Supplementary Figure S1B) and hence less deformable cells. Moreover, the assembly of new actin filaments was impaired that further leads to a less entangled actin filament network.

## Discussion

**FIGURE 9:**
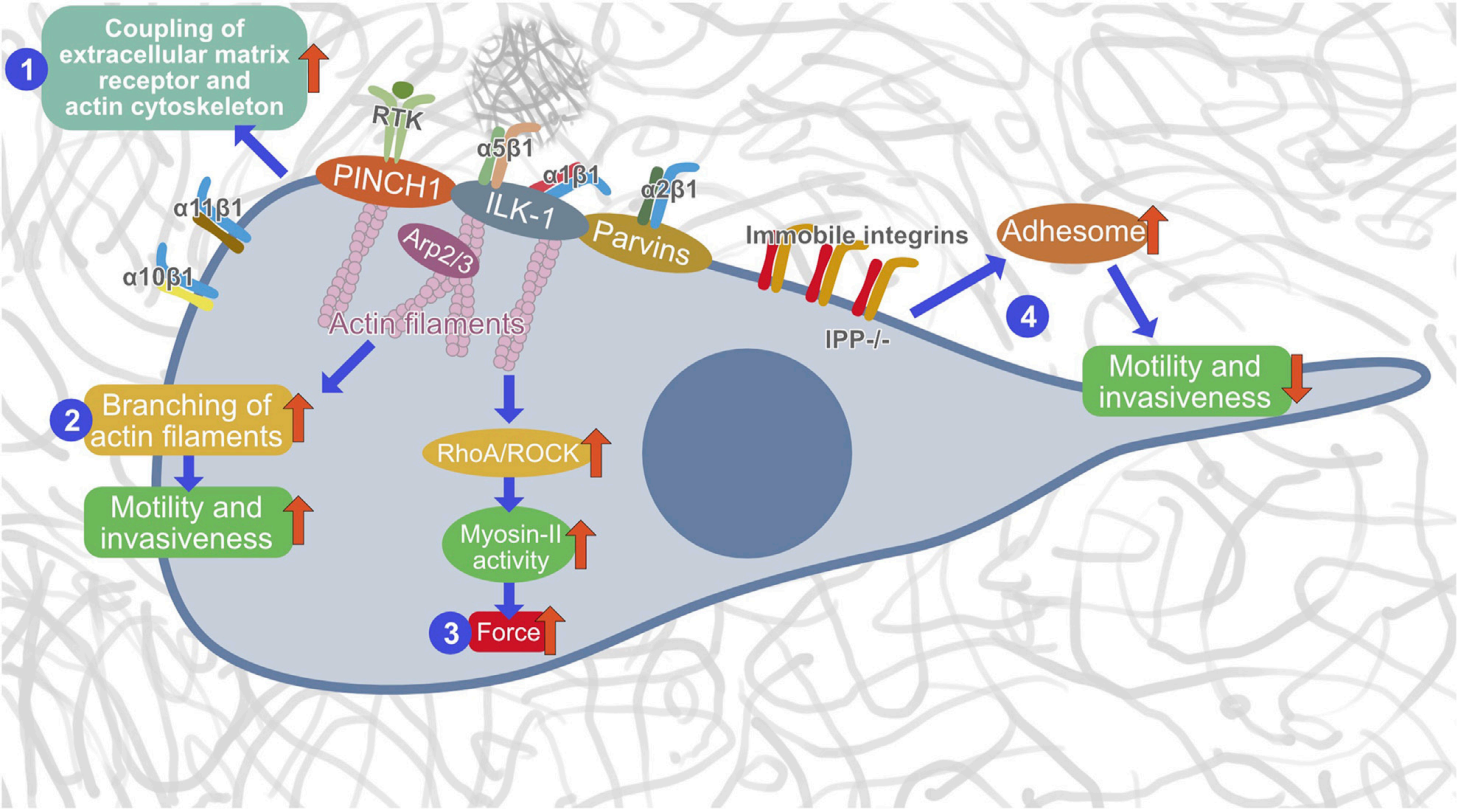
Hypothetical model of how PINCH1 acts in the IPP complex. There are three distinct functions of PINCH1, such as actin polymerization (1), generation of contractile forces (2) and promoting migration and invasion (3) through Arp2/3 driven branching of actin filaments. In the absence of an IPP complex, the integrins become immobile, the adhesome is strengthened and the migration and invasion capacity is reduced (4).

Dynamic coupling between IACs, such as focal adhesions and invadosomes (podosomes and invadopodia), and actin filaments is essential for upstream adhesion regulation. Notably, ILK-1 has been identified to enter the IPP complex that appears at focal adhesions prior to their localization (Zhang et al., 2002b). ILK’s binding to PINCH1 is a requirement to anchor to integrin-rich focal adhesions. Specifically, by recruiting the focal adhesion adaptors PINCH1 and Parvin into the heterotrimeric IPP complex, ILK-1 can trigger the bundling of F-actin filaments, a cognate process well known to generate a force/mechanical cue that encourages cytoskeletal reconstruction and dynamic cell adhesion (Vaynberg et al., 2018). In-depth structural analysis identified that PINCH1’s LIM1 domain can be coupled to the N-terminal ankyrin repeat domain (ARD) of ILK-1 (Velyvis et al., 2001; Chiswell et al., 2008; Yang et al., 2009; Stiegler et al., 2013). However, Parvin’s CH2 domain can tether to the C-terminal kinase-like domain (KLD) of ILK-1 (Fukuda et al., 2009; Fukuda et al., 2011; Stiegler et al., 2013). Consequently, both contributes to the establishment of a dense IPP complex (Fukuda et al., 2009). Thus, it can be assumed that PINCH1 functions within the IPP complex in coupling cell-matrix receptors to the cytoskeletal network. Therefore, we examined in this study the effect of PINCH1 on migration and invasion into dense 3D collagen matrices and found that silencing PINCH1 reduced MEF invasion into 3D collagen hydrogels and was associated with increased compliance, as measured by optical cell stretching. Although it has long been debated whether or not ILK-1 is a pseudokinase, because there was evidence that ILK-1 can phosphorylate a substrate *in vitro* (Hannigan et al., 2011), there seems to be a consensus that it is a pseudokinase. ILK-1 has a distinct ability to attach numerous proteins that govern cell adhesion and migration (Qin and Wu, 2012). Our PINCH1 results are consistent with our previous data that ILK-1 contributes to cell migration in a 3D collagen matrix which can be affected by inhibition of actin polymerization (Kunschmann et al., 2017). Therefore, we hypothesize that PINCH1 performs critical roles within the IPP complex by regulating the coupling of integrins to the actin filament network, promoting the bundling of actin and generation of forces that finally culminates in the promotion of cellular motility and invasiveness in the 3D extracellular matrix microenvironments (Figure 9). However, this model of PINCH1’s functions still needs further investigations.

Interestingly, knocking out of PINCH1 increased the compliance in the long axis upon laser-induced stretching, whereas the deformation in the short axis decreases, suggesting a change in the proxy for the Poisson’s ratio or connectivity of the cytoskeletal network of the cells by knocking out PINCH1. Thus, we have revealed that PINCH1 functions in providing cellular mechanical characteristics of fibroblasts that may foster their invasive capacity into 3D extracellular matrix environments. Subsequently, we investigated whether cytoskeletal modulations have differential effects in PINCH1^fl/fl^ cells and PINCH1 knock-out MEFs. Preventing F-actin polymerization with Latrunculin A results in a reduction of invasion in PINCH1^fl/fl^ cells and a further decrease in invasion beyond that induced by silencing PINCH1 in PINCH1 null MEFs. Perhaps interestingly, Latrunculin A treatment has a similar effect to PINCH1 knock-out in that there is a disconnect between the long and short axis deformation between PINCH1^fl/fl^ cells and treated cells, as was observed when PINCH1 was knocked out. This result indicates that there may be differences in the actin cytoskeletal network of the two cell types. However, application of Latrunculin A to PINCH1 knock-out MEFs results in an increase in both short and long axis deformation, which indicates a change in the cytoskeletal network. In fact, we have seen a change in the calculated proxy for the Poisson’s ratio. Inhibition of non-muscle myosin IIA also attenuated invasion but decreased compliance. Hence, the invasion relies on myosin activity in both cell types. The elevated compliance after myosin activity inhibition may indicate that the actin-myosin interaction is somehow stalled and myosin filaments function as actin crosslinkers, which may explain the increase in compliance.

PINCH1 is predestined to perceive the mechanical signals from the environment and due to the molecular structure and especially the structural properties of the domains. It can be hypothesized that the LIM domains of PINCH1 function as mechanosignal memories, which could render the PINCH1 protein a specialized protein that encodes the amount of mechanical stretch within its structural domains, which are more or less stretched and thus deformed. Beyond this storage of mechanical cues, PINCH1 has been found to couple to Rsu-1 to activate Rac1, and activation of Rac1 is required for cell propagation (Xu et al., 2016). These data suggest that specific domains of PINCH1 control two separate autonomous paths: one uses ILK-1 to enable cell adhesion, and the other enlists Rsu-1 to activate Rac1 to enhance cell propagation (Xu et al., 2016). This is supported by a recent finding of our group that knocking out of Rac1 impairs their migration and invasion into dense 3D collagen matrices (Kunschmann et al., 2019; Mierke et al., 2020). In fact, we have shown here that knock-out of PINCH1 let to impaired cellular motility in dense 3D collagen matrices, which demonstrates that it fulfills an important role. This is not at all inconsistent with the fact that a crucial mechanism for ILK has been uncovered, highlighting its uniqueness as a pseudokinase that transduces a non-catalytic signal and governs the adhesion of cells (Vaynberg et al., 2018).

We have revealed that inhibition of Arp2/3 increased invasive depth in PINCH1^fl/fl^ cells, but decreased invasion in PINCH1 knock-out MEFs, indicating an interaction between PINCH1 and Arp2/3 function. The stretching of both cell types showed that during inhibition of the Arp2/3 complex in PINCH1^fl/fl^ cells increased their deformability at both stretching conditions, whereas the stretching of PINCH1^−/−^ cells pronouncedly decreased cellular deformability indicating that the Arp2/3 complex is important for the stretching behavior of the cells. This result indicates that Arp2/3 contributes to the mechanophenotype of the cells.

However, these data do not allow us to conclude whether PINCH1 or ILK-1 is the favorite molecule in the IPP complex that provides its overall function. We assume that the components of the IPP complex experience conformational alterations upon force application in order to convey forces within the integrin adhesions (Vakaloglou et al., 2016). It is well-known that the ILK-1 ankyrin repeat domain exhibits spring-like characteristics and hence elastic properties and thereby can react to forces by unfolding of its own protein domains (Lee et al., 2006). In line with these results, quantitative proteomic analysis of mechanotransduction pathways in mammalian cells has identified that LIM domain proteins, such as PINCH1, which possesses five LIM domains, fulfill tasks as potential tension sensors (Schiller et al., 2011; Anderson et al., 2021). Numerous proteins possessing a LIM domain exhibit reduced integrin-based focal adhesion or stress fiber recruitment upon actomyosin contractility inhibition (Kuo et al., 2011; Schiller et al., 2011), implying that the LIM domains may act as a mechanical reaction unit. Mechanoaccumulation has also been noted in distinct members of the paxillin family of focal adhesion proteins (Smith et al., 2013; Watanabe-Nakayama et al., 2013), but it is not clear how widespread this activity is among the LIM protein superfamily.

For determination of the elastic modulus and the viscosity, we employed the Kelvin-Voigt model. However, there is a need for this model to be adapted, since the elastic modulus and viscosity depend on one another. Due to the versatile mechanical properties of cells, there are rheological aspects that cannot be effectively captured by this model. Our findings suggest that further empirical effort is needed to obtain detailed quantitative information on the mechanical properties of the cells in various inhibitor treatments in order to build mechanical models with stress-strain constitutive equations for the cell that more faithfully represent the underlying cell rheology.

Future investigations of the nanomechanical characteristics of ILK ankyrin repeats and LIM domain proteins, such as PINCH1, will be informative. Our findings indicate that fibroblasts preferentially use IPP complex proteins, such as PINCH1 or ILK-1, to fulfill migratory tasks and to maintain the cellular mechanophenotype. Based on these findings and our previous results on ILK-1, we conclude that the IPP complex fulfills a crucial function in providing a mechanical phenotype of cells. Moreover, we hypothesize that this complex is also involved in sensing, transducing and “coding” (storage) of mechanical signals (external inputs).

In conclusion, our results that PINCH1 knockout cells were less invasive and more deformable compared to controls indicate that PINCH1 fulfills a crucial role. Hence, we propose a new model of the functional role of PINCH1 within the IPP complex from a biophysical viewpoint (Figure 9). These findings on PINCH1 provide a valuable resource to advance our understanding of the participation of PINCH1 in contributing to the mechanical properties of fibroblast and cellular motility in 3D collagen matrices.

## Key Findings (Impact on Science)

• PINCH1 knockout reduces fibroblast invasion into 3D collagen hydrogels
• PINCH1 knockout increases the deformability and reduces elastic modulus of cells
• PINCH1 knockout elevates compliance in the long axis of the laser-based stretch exertion, but a decrease in short axis deformation
• Latrunculin A applied to PINCH1 knockout MEFs causes enhanced short and long axis deformation
• Inhibition of Arp2/3 impairs the invasion in PINCH1 knockout MEFs, but elevates the invasion depths in PINCH1 control cells, indicating an interplay between Arp2/3 function and PINCH1
• PINCH1 is not merely an adaptor protein, it is essential for the IPP complex to provide cell mechanical characteristics

## Materials and Methods

### Optical Cell Stretcher Measurement and Data Acquisition

For each optical cell stretcher experiment, cells were prepared as described in (Mierke, 2019; Mierke et al., 2020). PINCH1^fl/fl^ and PINCH1^−/−^ cells were cultured at 37°C and 5% CO_2_ to 70%–80% confluency in a T25 cell culture flask and subsequently harvested by Trypsin/EDTA induced dislodging. Resuspended single cells were directly measured using a commercially available, automated Optical Cell Stretcher device for 3–5 h (RS Zelltechnik, Leipzig, Germany). This device traps suspended single cells in a dual beam laser trap at 100 mW for 1 s, subsequently stretches the individual cell at a selected “low” laser power of 800 mW or a selected “high” laser power of 1,200 mW, and finally lets the stretched cell relax at the trap laser power of 100 mW, see (Figures 2A–D). These two laser powers were selected after analyzing a power range from 500 to 1,200 mW in steps of 100 mW. Specifically, we have measured single cells of the same cell sample pool randomly (performed by the acquisition program) at either 800 mW or 1,200 mW to reduce experiment-to-experiment variations. This results in 5 s videos (1 s trap, 2 s stretch, 2 s relaxation) that enable a detection of the cell boundary and further automatically analysis. For each prepared sample, typically 1,500–6,000 cells were recorded, whereby generally 1,500–2,500 cells were measured per single experiment. We verified that there were no differences between the initial 500 cells measured and the final 500 cells measured (Supplementary Figure S3). The experiments were carried out in quadruplicate or quintuplicate. Specifically, optical cell stretcher experiments were performed with buffer-treated (vehicle control) cells or cells treated with 0.4 μM Latrunculin A (Sigma Aldrich, Taufkirchen, Germany), 100 μM CK666 (Sigma Aldrich) or 100 μM Blebbistatin-treated (Merck, Darmstadt, Germany) 2 h prior to the start of measurement.

### Optical Cell Stretcher Data Analysis

The Optical Cell Stretcher analysis software automatically diminishes cell rotation and calculates several parameters, such as cell deformation along major and minor axes, and stores this data in JSON format files. These files contain metadata such as the randomly chosen laser power as well as vector data of frame-time and corresponding time-dependent parameters such as long axis deformation. This data was further processed using custom made Python programs. Vector data was used to plot time-dependent graphs, such as long axis deformation. For this, the median stretch curve with corresponding 95% (unless otherwise stated) confidence interval was calculated and plotted in dependence on elapsed time, as seen in Figures 2A–D,I–L. The vector data of individual cells were further analyzed by extracting values at the end of each stretch phase. This yields parameters such as maximum cell deformation, as seen in Figures 2E–H,M–P. The large number of recorded cells for each individual measurement enables statistically relevant and highly reproducible results.

### 3D Collagen Hydrogel Invasion Assays

Cell invasion experiments were prepared and carried out as described in (Fischer et al., 2017; Mierke et al., 2017; Kunschmann et al., 2019). Collagen I monomers extracted from rat tail (4 mg/ml rat collagen type I, Serva, Heidelberg, Germany) and bovine skin (4 mg/ml bovine collagen type I, Biochrom, Berlin, Germany) in acidic solution at 0°C were mixed in a mass fraction of 1:2, respectively. A 1M phosphate buffered solution at 0°C was added such that the final solution has a pH value of 7.4, ionic strength of 0.7, phosphate concentration of 200 mM, and a specific monomer concentration, such as 1.5 g/l or 3.0 g/l. Subsequently, 1.2 ml of the cooled solution was put in each well of a 6-well plate (Greiner Bio-One, Frickenhausen, Germany) and polymerized in an incubator at 37°C, 5% CO_2_ and 95% relative humidity. The polymerized collagen matrices were rinsed three times with PBS and incubated with 2 ml cell culture medium overnight. Cells were grown in a T25 cell culture flask and harvested at 70%–80% confluency. Subsequently, 50,000 cells were resuspended in 2 ml cell culture medium. The collagen gels were rinsed, and the suspended cell solution was put on top of each collagen gel and placed in an incubator at 37°C, 5% CO_2_, 95% humidity. After 12 h incubation time, the collagen gels were rinsed and 2 ml fresh cell culture medium with dissolved pharmacological drug or vehicle control was added for another 72 h at 37°C, 5% CO_2_, 95% humidity. Finally, the gels were fixed using 2.5% glutaraldehyde for 20 min in an incubator, rinsed three times with PBS, and cell nuclei were stained using 4 μg/ml HOECHST 33342 solution.

### Invasion Data Analysis

The samples of invaded cells with stained nuclei in collagen gels were imaged utilizing epifluorescence. This image stacks recorded with a custom-built setup based on a DMI6000B microscope (Leica; Wetzlar, Germany), as described in (Fischer et al., 2020; Hayn et al., 2020).

However, in this publication, we have improved our invasion analysis technique to allow data collection even with a 10× objective. This now allows us to analyze a far greater number of cells in a shorter time, significantly shortening data acquisition times. The custom-built analysis algorithm as described in (Fischer et al., 2020; Hayn et al., 2020) has been improved to analyze images recorded with a 10× objective to enable the analysis of somewhat larger image areas while keeping image resolutions constant. Only minor adjustments had to be made to the analysis algorithm because the size of the fields of interest was different due to a change in the conversion factor from pixels to micrometers. The recorded image stacks have a z-resolution of 4 μm and are further processed as described in (Fischer et al., 2020; Hayn et al., 2020) to yield invasiveness and invasion depth of the analyzed cell populations. The selected z-resolution of 4 μm, which is approximately the size of a nucleus, is sufficient to analyze cell invasion. The analysis is independent of background translucency and can accurately determine the z-position by using a focus measure to find the exact z-position.

The invasion profile referred to in the manuscript represents the cumulative probability on cell numbers dependent on the depth of the gel, as published earlier (Mierke et al., 2010; Mierke et al., 2011; Mierke et al., 2017). This so-called invasion profile shows the probability at a certain depth to find cells below this certain depth. This is an excellent measure to compare depth-dependent invasiveness independent of total and relative invasion cell numbers, where often differently skewed distributions complicate a visual comparison. Due to the vast amounts of cells used in the calculations done here, the invasion profile predominantly may appear smoothed. However, as an accumulated dataset per condition is used, no averaging is performed and thus no error bars can be provided.

### Modulation of Cell Invasion Through Pharmacological Inhibitory Substances

To inhibit or modulate cell invasion, we added 0.4 μM Latrunculin A (actin polymerization inhibitor), 100 μM Blebbistatin (myosin II inhibitor), 100 μM CK666 (Arp2/3 inhibitor) to the collagen invasion assay 12 h after cell seeding for the invasion assay. The total duration of the invasion assay was three days plus 12 h preincubation. Then, the cells are treated and analyzed as described above.

### Fluorescence Microscopic Analysis Using a Confocal Laser Scanning Microscope

Applied to cell stimulation with inhibitory drugs: For appropriate cell adhesion, the purified glass slides were layered with various extracellular matrix proteins such as 10 μg/ml laminin for 2 h at 37°C. To eliminate the uncoated extracellular matrix proteins, the coated slides were rinsed twice with PBS. 3,000 to 6,000 cells per square centimeter were seeded and grown for 16 h either in the absence (vehicle control: DMSO buffer unless otherwise stated) or in the presence of 0.4 μM Latrunculin A, 100 μM Blebbistatin or 100 μM CK666 for 2 h at 37°C. To finish and further analyze the inhibitory drug-stimulated cells, cells are fixated with 4% oaraformaldehyde, rinsed at least twice with PBS, permeabilized with 0.1% TritonX100 for 5 min at room temperature, rinsed, and incubated overnight with 1% BSA in PBS. To visualize the cytoskeleton, nucleus and cell membrane, cells are stained with 10 units/ml Alexa Fluor 546 conjugated phalloidin dissolved in 1% BSA in PBS, 0.25 mg/ml DiD and 0.02 mg/ml 33342 Hoechst at 4°C overnight. For decreasing fading, slides are prepared with prolong gold antifade and placed on a standard microscopic glass slide. They are left to incubate for 24 h at 4°C to yield a gel-like antifade, sealed with nitrocellulose lacquer, and analyzed directly using a laser scanning microscope by taking image stacks (TCS SP8, Leica, Wetzlar, Germany). Experiments have been rerun three times independently, and 20–30 cells have been imaged per each condition and staining.

### Statistical Analysis

All experimental cell invasion data were expressed as box and whiskers plots. A boxplot is a form of visualization of distributions of data based on a five-number summary, minimum and maximum, median and first and third quartiles. The top and bottom edges of the box represent the Q1 or 25th percentile and Q3 or 75th percentile, respectively. The middle line represents the median or Q2 or 50th percentile. Whiskers denote the lines that run above and below the box. The first step in determining the whiskers is to calculate the interquartile range (IQR) which is IQR = Q3-Q1. The upper and lower ends of the whiskers are usually in a distance from the box of 1.5*IQR, so Q3+1.5*IQR and Q1-1.5*IQR. The ends constitute the minimum and maximum of our data set. Any data point below or above the whisker ends is considered an outlier.

The invasion profiles are presented as cumulative distributions. These data were bootstrapped to allow hypothesis testing for otherwise singular distribution values such as depth of invasion and invasiveness of a cell population. All experimental cell deformability data were expressed as median and 95% confidence interval. Statistical analyses were conducted using the nonparametric Mann-Whitney U test and the Kruskal-Wallis test for non-normal distributions and unequal variances. They are provided as Supplementary Tables S1, S2 for deformability data and as Supplementary Tables S3, S4 for percentage of invasive cell and invasion depths data, respectively (see Supplementary Material). In addition, we carried out Bonferroni corrections for each hypothesis to further enhance the statistical power of our analysis. In general, *p*-values of ≤ 0.05 were deemed statistically significant and marked with one asterisk, *p*-values of ≤ 0.01 were highlighted with two asterisks, and *p*-values of ≤ 0.001 were highlighted with three asterisks.

## Data Availability

The raw data supporting the conclusion of this article will be made available by the authors, without undue reservation.
