# Supplementary material for: PINCH1 Promotes Fibroblast Migration in Extracellular Matrices and Influences Their Mechanophenotype
Source: Front Cell Dev Biol. 2022 May 16;10:869563. doi: 10.3389/fcell.2022.869563 (PMC9149598; doi:10.3389/fcell.2022.869563)
Supplement: Supplementary file 1 [file DataSheet1.pdf]

Supplementary Material

Supplementary Figure 1. Elastic modulus and viscosity of PINCH1$^{-/-}$ and PINCH1$^{fl/fl}$ cells in the presence of DMSO (control) or inhibitory drug, such as 0.4 µM Latrunculin A, 100 µM Blebbistatin or 100 µM CK666., based on the application of the Kelvin-Voigt model on the optical cell stretcher data.

A

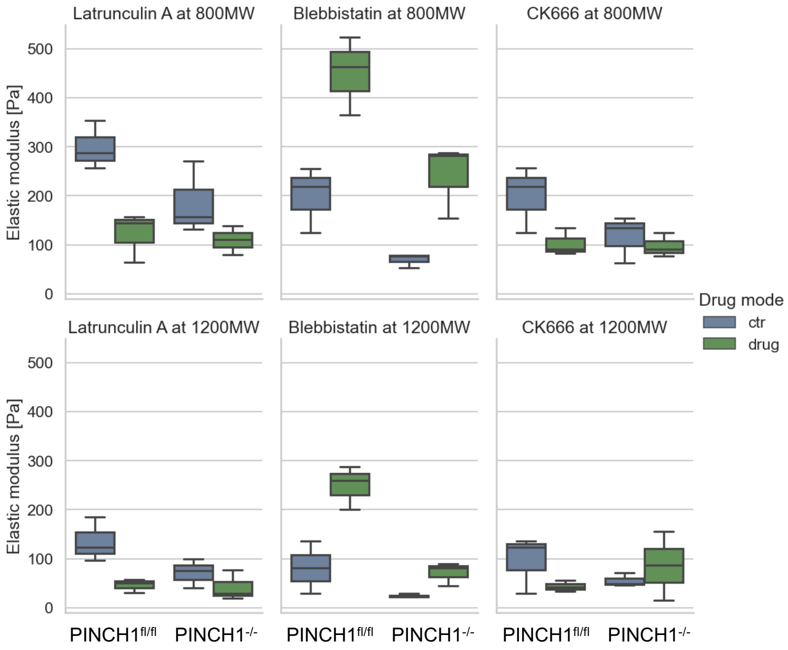

B

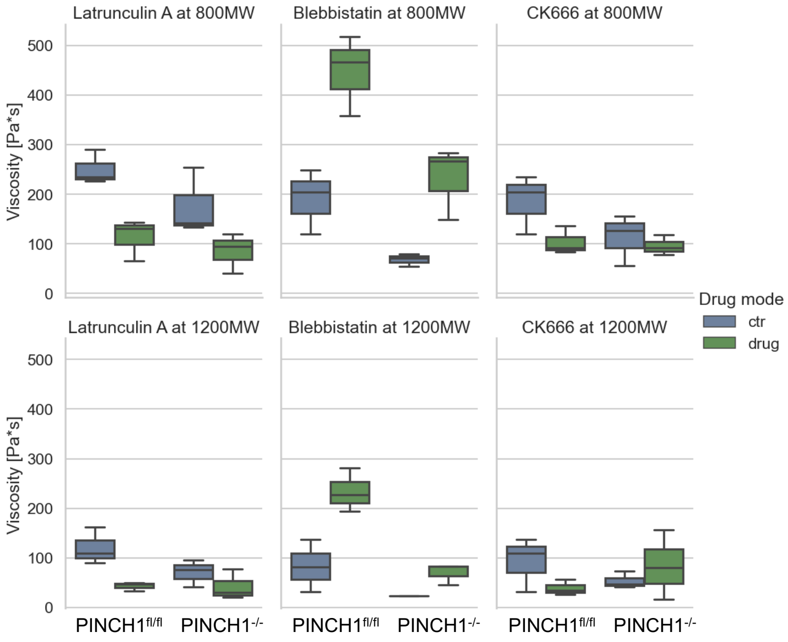

Supplementary Figure 2. The Proxy for Poisson ratio (short axis over long axis of figure 4 in the manuscript) is provided for PINCH1$^{-/-}$ cells and PINCH1$^{fl/fl}$ cells in the presence of DMSO (control, blue) or Latrunculin A (orange). The data are presented as median values ± SD.

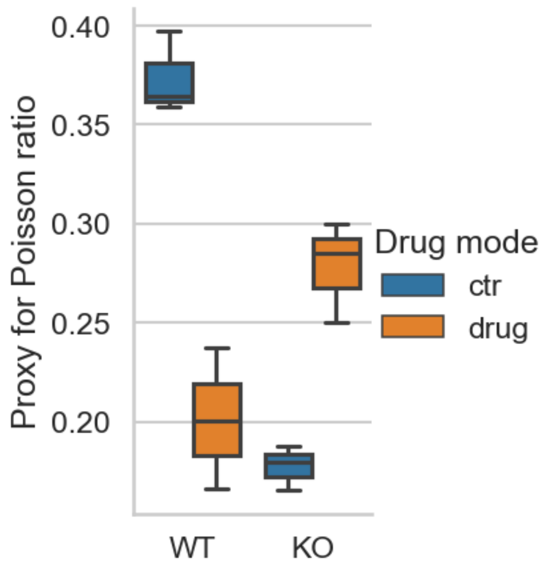

Supplementary Figure 3. The Proxy for the Poisson ratio has been plotted for the first 500 measured cells and the last 500 cells of the optical deformability analysis in the presence of pharmaceutical drugs, such as 0.4 µM Latrunculin A, 100 µM Blebbistatin and 100 µM CK666. There are no significant changes in the Proxy for the Poisson ratio of the 0-500 measured cells (initial phase of the analysis) and the 500-1000 measured cells (final phase of the analysis) for the different conditions.

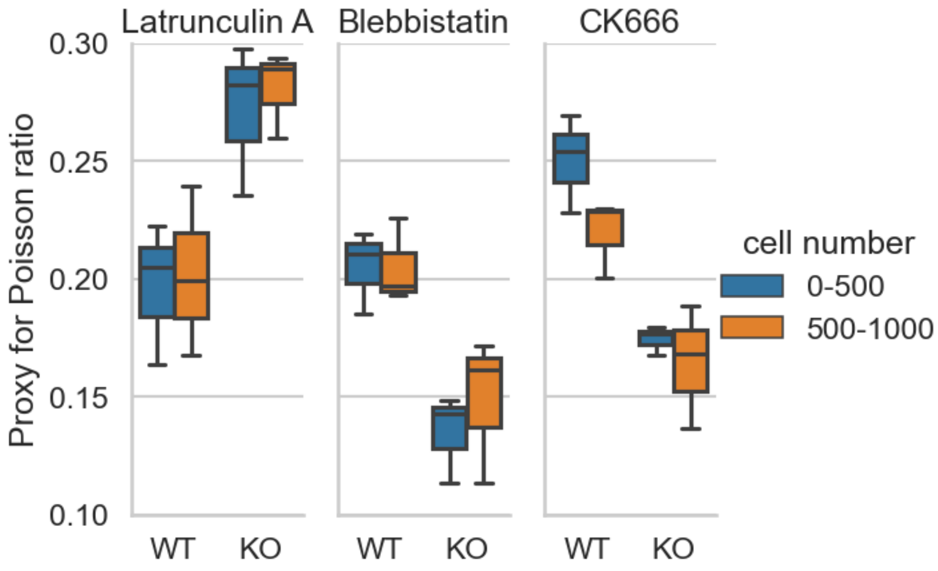

Supplementary Table 1. Statistical analysis of the optical cell stretcher data using the Kruskal-Wallis test.

Drug	Laser power	Value	p-value	Statistics	Significance
Blebbistatin	800	Long axis deformation EoS [%]	6,7595E-135	623,8636134	***
		Short axis deformation EoS [%]	5,8753E-69	319,5367488	***
	1200	Long axis deformation EoS [%]	5,3925E-173	799,5594071	***
		Short axis deformation EoS [%]	1,43292E-18	86,20155686	***
Latrunculin A	800	Long axis deformation EoS [%]	1,11415E-70	327,4916915	***
		Short axis deformation EoS [%]	4,10549E-40	186,1678787	***
	1200	Long axis deformation EoS [%]	1,1674E-275	1272,81128	***
		Short axis deformation EoS [%]	1,63596E-78	363,6689473	***
CK666	800	Long axis deformation EoS [%]	7,80689E-46	212,6451962	***
		Short axis deformation EoS [%]	2,37121E-28	131,6616406	***
	1200	Long axis deformation EoS [%]	2,1312E-105	487,7718834	***
		Short axis deformation EoS [%]	2,8177E-09	42,72311956	***

Supplementary Table 2. Statistical analysis of the optical cell stretcher data using Mann-Whitney-U test.

drug	laser power	value	sample a	sample b	p-value	statistic	significance
Blebbistatin	800	Long axis deformation EoS [%]	WT Blebb ctr 100µM	WT Blebb 100µM	4,18693E-37	1681381,5	***
		Long axis deformation EoS [%]	KO Blebb ctr 100µM	KO Blebb 100µM	1,37452E-47	1823379	***
		Long axis deformation EoS [%]	WT Blebb ctr 100µM	KO Blebb ctr 100µM	4,76454E-36	1095942	***
		Long axis deformation EoS [%]	WT Blebb 100µM	KO Blebb 100µM	7,89807E-31	993989,5	***
		Short axis deformation EoS [%]	WT Blebb ctr 100µM	WT Blebb 100µM	5,95099E-35	1004248	***
		Short axis deformation EoS [%]	KO Blebb ctr 100µM	KO Blebb 100µM	0,000187371	1309829	***
		Short axis deformation EoS [%]	WT Blebb ctr 100µM	KO Blebb ctr 100µM	5,9788E-40	1075980	***
		Short axis deformation EoS [%]	WT Blebb 100µM	KO Blebb 100µM	0,000308196	1203724,5	**
	1200	Long axis deformation EoS [%]	WT Blebb ctr 100µM	WT Blebb 100µM	3,09875E-25	1505401,5	***
		Long axis deformation EoS [%]	KO Blebb ctr 100µM	KO Blebb 100µM	4,44412E-33	1763223	***
		Long axis deformation EoS [%]	WT Blebb ctr 100µM	KO Blebb ctr 100µM	4,31613E-86	881423	***
		Long axis deformation EoS [%]	WT Blebb 100µM	KO Blebb 100µM	1,98875E-55	826849,5	***
		Short axis deformation EoS [%]	WT Blebb ctr 100µM	WT Blebb 100µM	2,43088E-09	1087980	***
		Short axis deformation EoS [%]	KO Blebb ctr 100µM	KO Blebb 100µM	0,725676119	1414040,5	n.s.
		Short axis deformation EoS [%]	WT Blebb ctr 100µM	KO Blebb ctr 100µM	1,28094E-15	1215255	***
		Short axis deformation EoS [%]	WT Blebb 100µM	KO Blebb 100µM	0,092393371	1180593	n.s.
Latrunculin A	800	Long axis deformation EoS [%]	WT LatA ctr 0.4µM	WT LatA 0.4µM	5,072E-31	1024622,5	***
		Long axis deformation EoS [%]	KO LatA ctr 0.4µM	KO LatA 0.4µM	0,03286488	1285218	n.s.
		Long axis deformation EoS [%]	WT LatA ctr 0.4µM	KO LatA ctr 0.4µM	1,6889E-45	933167,5	***
		Long axis deformation EoS [%]	WT LatA 0.4µM	KO LatA 0.4µM	2,08995E-10	1196737	***
		Short axis deformation EoS [%]	WT LatA ctr 0.4µM	WT LatA 0.4µM	1,57814E-29	1032682	***
		Short axis deformation EoS [%]	KO LatA ctr 0.4µM	KO LatA 0.4µM	1,28724E-14	1551865,5	***
		Short axis deformation EoS [%]	WT LatA ctr 0.4µM	KO LatA ctr 0.4µM	1,27941E-23	1043218,5	***
		Short axis deformation EoS [%]	WT LatA 0.4µM	KO LatA 0.4µM	6,06701E-19	1616361,5	***
	1200	Long axis deformation EoS [%]	WT LatA ctr 0.4µM	WT LatA 0.4µM	6,96483E-90	860144	***
		Long axis deformation EoS [%]	KO LatA ctr 0.4µM	KO LatA 0.4µM	1,65458E-37	1047868	***
		Long axis deformation EoS [%]	WT LatA ctr 0.4µM	KO LatA ctr 0.4µM	1,4742E-113	724220,5	***
		Long axis deformation EoS [%]	WT LatA 0.4µM	KO LatA 0.4µM	4,63431E-82	941358,5	***
		Short axis deformation EoS [%]	WT LatA ctr 0.4µM	WT LatA 0.4µM	8,29752E-08	1279085,5	***
		Short axis deformation EoS [%]	KO LatA ctr 0.4µM	KO LatA 0.4µM	2,34852E-59	1863519,5	***
		Short axis deformation EoS [%]	WT LatA ctr 0.4µM	KO LatA ctr 0.4µM	2,11726E-10	1163865	***
		Short axis deformation EoS [%]	WT LatA 0.4µM	KO LatA 0.4µM	5,6677E-54	1965934,5	***
CK666	800	Long axis deformation EoS [%]	WT CK666 ctr 100µM	WT CK666 100µM	4,03124E-36	1173200	***
		Long axis deformation EoS [%]	KO CK666 ctr 100µM	KO CK666 100µM	0,001162329	1562888	**
		Long axis deformation EoS [%]	WT CK666 ctr 100µM	KO CK666 ctr 100µM	2,81094E-35	1084973	***
		Long axis deformation EoS [%]	WT CK666 100µM	KO CK666 100µM	0,008331728	1666148	*
		Short axis deformation EoS [%]	WT CK666 ctr 100µM	WT CK666 100µM	0,042872679	1491304	n.s.
		Short axis deformation EoS [%]	KO CK666 ctr 100µM	KO CK666 100µM	0,000393634	1366097	**
		Short axis deformation EoS [%]	WT CK666 ctr 100µM	KO CK666 ctr 100µM	4,27835E-12	1240909,5	***
		Short axis deformation EoS [%]	WT CK666 100µM	KO CK666 100µM	1,52169E-16	1331794,5	***
	1200	Long axis deformation EoS [%]	WT CK666 ctr 100µM	WT CK666 100µM	1,01879E-10	1285452	***
		Long axis deformation EoS [%]	KO CK666 ctr 100µM	KO CK666 100µM	1,08619E-16	1645724	***
		Long axis deformation EoS [%]	WT CK666 ctr 100µM	KO CK666 ctr 100µM	8,86612E-89	915365	***
		Long axis deformation EoS [%]	WT CK666 100µM	KO CK666 100µM	4,80169E-13	1182318	***
		Short axis deformation EoS [%]	WT CK666 ctr 100µM	WT CK666 100µM	6,42397E-08	1316160	***
		Short axis deformation EoS [%]	KO CK666 ctr 100µM	KO CK666 100µM	0,02507332	1349185	n.s.
		Short axis deformation EoS [%]	WT CK666 ctr 100µM	KO CK666 ctr 100µM	0,000135056	1392128,5	***
		Short axis deformation EoS [%]	WT CK666 100µM	KO CK666 100µM	0,589600539	1367647	n.s.

Supplementary Table 3. Statistical analysis of the percentage of invasive cell data using Mann-Whitney-U test.

	value	drug	p-value	statistic	significance
**0**	Invasive cells [%]	Blebbistatin	1,01E-11	54,212	***
**1**	Invasive cells [%]	Latrunculin A	5,82E-11	50,64564	***
**2**	Invasive cells [%]	CK666	7,06E-12	54,94295	***

Supplementary Table 4. Statistical analysis of the invasion depth data using Mann-Whitney-U test.

	value	drug	p-value	statistic	significance
**0**	Invasion depth [µm]	Blebbistatin	8,74E-12	54,50881	***
**1**	Invasion depth [µm]	Latrunculin A	3,44E-12	56,40775	***
**2**	Invasion depth [µm]	CK666	1,01E-11	54,21199	***
